# Actomyosin-Driven Tension at Compartmental Boundaries Orients Cell Division Independently of Cell Geometry *In Vivo*

**DOI:** 10.1016/j.devcel.2018.10.029

**Published:** 2018-12-17

**Authors:** Elena Scarpa, Cédric Finet, Guy B. Blanchard, Bénédicte Sanson

**Affiliations:** 1 Department of Physiology, Development and Neuroscience, University of Cambridge, Downing Street, Cambridge CB2 3DY, UK

**Keywords:** embryogenesis, morphogenesis, *Drosophila*, cell division orientation, tissue homeostasis, quantitative developmental biology, actomyosin cortex, cell mechanics

## Abstract

Cell shape is known to influence the plane of cell division. *In vitro*, mechanical constraints can also orient mitoses; however, *in vivo* it is not clear whether tension can orient the mitotic spindle directly, because tissue-scale forces can change cell shape. During segmentation of the *Drosophila* embryo, actomyosin is enriched along compartment boundaries forming supracellular cables that keep cells segregated into distinct compartments. Here, we show that these actomyosin cables orient the planar division of boundary cells perpendicular to the boundaries. This bias overrides the influence of cell shape, when cells are mildly elongated. By decreasing actomyosin cable tension with laser ablation or, conversely, ectopically increasing tension with laser wounding, we demonstrate that local tension is necessary and sufficient to orient mitoses *in vivo*. This involves capture of the spindle pole by the actomyosin cortex. These findings highlight the importance of actomyosin-mediated tension in spindle orientation *in vivo*.

## Highlights

• Parasegmental boundary cells (PSB) do not divide according to interphase shape
• PSB actomyosin cables are essential to orient mitoses perpendicular to the boundary
• Cable laser ablation demonstrates that anisotropic tension orients cell divisions
• PSB mitoses are oriented by capture of the centrosome by the actomyosin cortex

## Graphical Abstract

**Figure:**
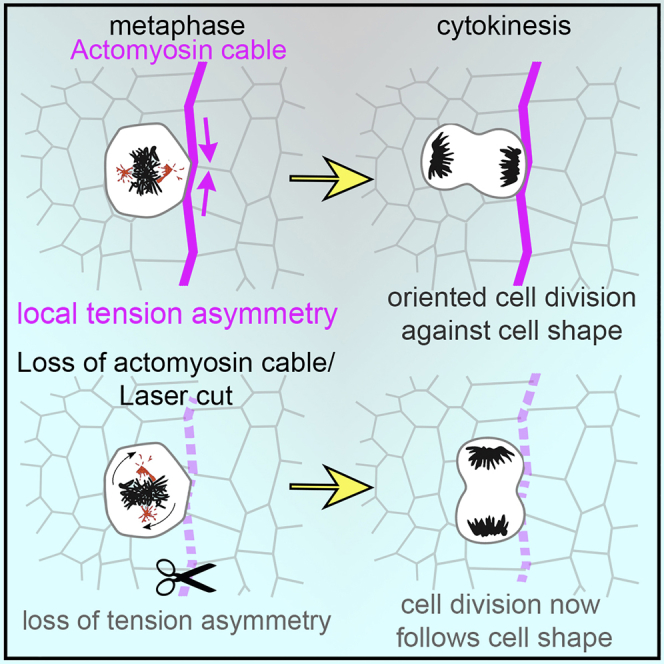

## Teaser

Scarpa et al. investigated whether mechanical forces can control the orientation of the mitotic spindle *in vivo*. They found that in the *Drosophila* embryo, an actomyosin cable, rather than cell shape, orients mitoses at compartmental boundaries. Higher tension along this structure promotes centrosome capture by the actomyosin cortex.

## Introduction

Regulation of the orientation of cell division is important for tissue morphogenesis, and if defective, can lead to disease such as tumorigenesis (McCaffrey and Macara, 2011), kidney malformations (Fischer et al., 2006), or microcephaly (Megraw et al., 2011). In developing epithelia, mitoses are usually oriented along the plane of the tissue, contributing to tissue elongation (da Silva and Vincent, 2007) or homeostasis (Campinho et al., 2013, Mao et al., 2013). The plane of cell division is given by the final position of the mitotic spindle, and animal cells tend to orient their mitotic spindle parallel to the longest axis of interphase cell shape (Hertwig, 1884, Minc et al., 2011, Tsou et al., 2003). Recently, the distribution of the tricellular vertices in the fly notum epithelium was found to be also a predictor of the orientation of the mitotic spindle and, in moderately elongated cells, a better predictor than interphase cell shape (Bosveld et al., 2016).

Recent work on isolated cells cultured on micropatterns has shown that physical forces may control the orientation of the mitotic spindle independently of cell shape (Fink et al., 2011). *In vivo*, it has been observed that tissue-level extrinsic forces can orient the mitotic spindle along the direction of stress (Campinho et al., 2013, Fink et al., 2011, Mao et al., 2013, Wyatt et al., 2015). However, because tissue-scale forces also cause planar cell elongation in epithelia (Butler et al., 2009, Lye et al., 2015, Mao et al., 2013, Wyatt et al., 2015), it has been difficult to disentangle a direct effect of forces from an indirect effect on cell geometry *in vivo* (Wyatt et al., 2015).

Here, we have discovered a population of cells in the *Drosophila* embryonic epidermis whose mitoses do not follow the long axis rule. These cells are located at the parasegmental boundaries (PSBs) and divide perpendicular to a contractile actomyosin cable that forms at the boundary cell-cell interfaces (Monier et al., 2010). We provide evidence that the orientation of the division plane of the boundary cells is governed directly by local tension anisotropy rather than by cell geometry or genetic cues.

## Results

### Cells Dividing at Parasegment Boundaries Do Not Follow the Long Axis Rule

**Figure 1:**
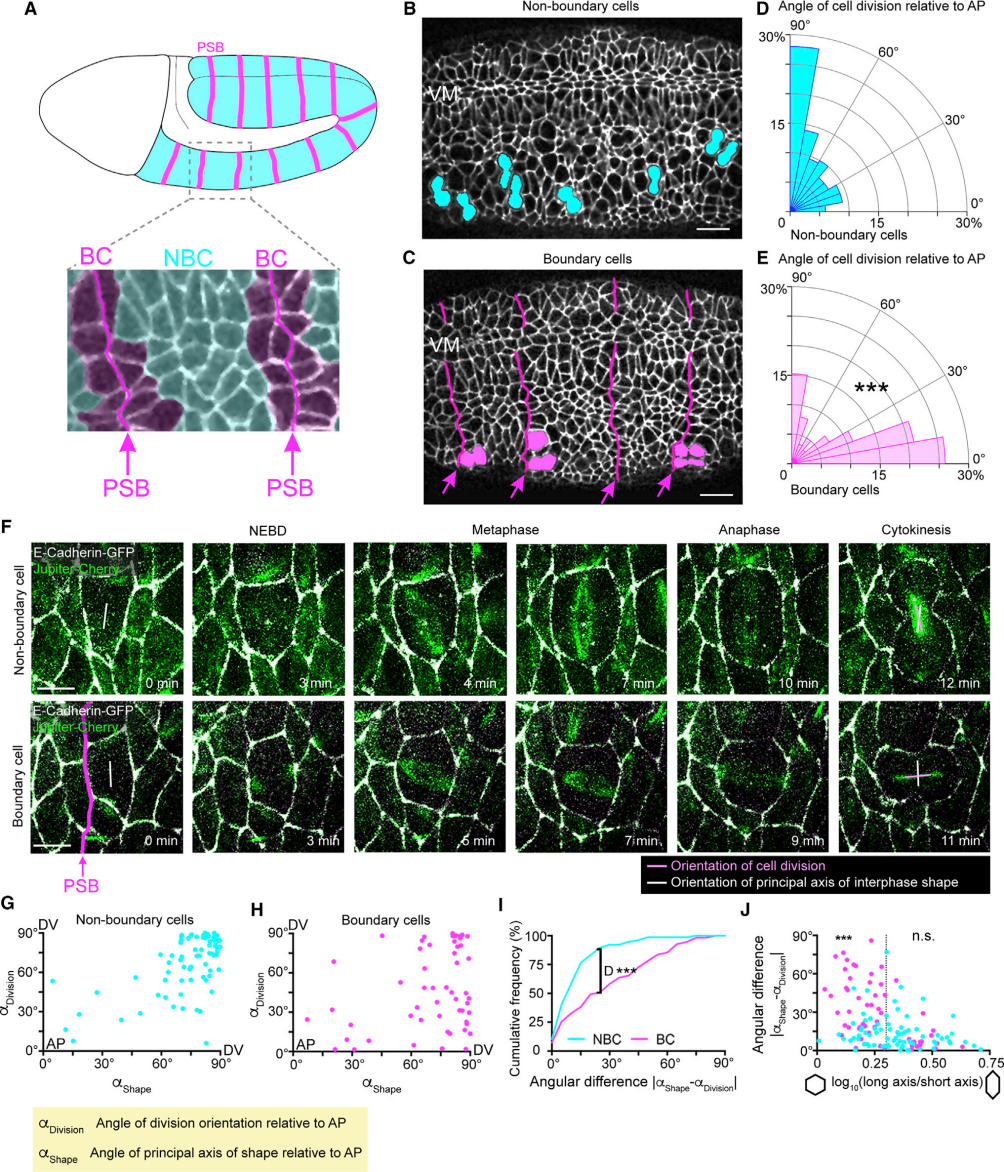
Cells Dividing at the Parasegment Boundary Do Not Follow the Long Axis Rule (A) Diagram of a *Drosophila* embryo when the germband (blue) is extended (stages 9 to 11). Cell divisions occur throughout the extended germband epidermis. The metameric subdivisions are the parasegments, separated by parasegment boundaries (PSBs, pink). BC, boundary cells; NBC, non-boundary cells. Examples of the planar cell division biases found in non-boundary (B) and boundary cells (C). VM, ventral midline. Scale bar, 10 μm. (D) Quantification of the angle of cell division in fixed embryos relative to the antero-posterior (AP) axis in NBC (n = 391 cell divisions) and BC (E) (n = 289 cell divisions; Mann-Whitney test, *U* = 34501, ^∗∗∗^p < 0.0001). Identification of the boundaries and strategy to measure division angles are described in Figures S1A and S1B, and STAR Methods. (F) Examples of dividing NBC and BC from time-lapse images of an *ubi-DE::Cadherin/en-Venus; jupiter::mCherry/+* embryo. *en-Venus* was used to identify PSBs (not shown) and *jupiter::mCherry* (green) to label the mitotic spindle. The orientation of cell division (pink vector) versus the orientation of interphase cell shape (white vector) is shown. Scale bar, 5 μm. (G) In NBC, there is a correlation between these two angles, suggesting that these cells follow the long axis rule (n = 77; Spearman’s rho test, *r* = 0.48, p < 0.001). (H) This correlation is absent for BC (n = 55; Spearman’s rho test, *r* = 0.17, p = 0.19). (I) Cumulative histogram for the angular difference between the orientation of cell division and the orientation of interphase cell shape for NBC (blue) and BC (magenta) (NBC, n = 77; BC, n = 55; Kolmogorov-Smirnov test, *D* = 0.41, ^∗∗∗^p < 0.001). (J) Angular difference between division and shape orientations as a function of log_10_ (long axis/short axis). For elongated cells (above 0.3, long axis/short axis ratio of 2), both NBC and BC behave similarly (NBC, n = 48; BC, n = 16; Kruskal-Wallis test, *H* = 39.58, n.s. p > 0.99), following the long axis rule. For moderately elongated cells (below 0.3) however, NBC and BC behaviors are significantly different (NBC, n = 29; BC, n = 39; Kruskal-Wallis test, *H* = 39.58, ^∗∗∗^p = 0.0003).

During *Drosophila* embryogenesis, the epidermis undergoes waves of cell divisions at extended germband stages 9 to 11 (Foe, 1989, Martinez-Arias, 1993). PSBs form through patterning mechanisms and prevent cells or their descendants from changing compartments (Monier et al., 2010, Vincent and O'Farrell, 1992) (Figure 1A). Here, we find that at these stages, boundary cells (BCs; cells with an edge contributing to a boundary) bias their orientation of division differently from non-boundary cells (NBCs) (Figures 1A–1C). Note that all angles are given relative to the antero-posterior (AP) axis throughout the manuscript (angle measurements are described in Figures S1A and S1B and STAR Methods). In fixed embryos, NBCs divide predominantly perpendicular to the AP axis of the embryo (Figures 1B and 1D). In contrast, BCs predominantly orient their divisions parallel to the AP axis of the embryo, perpendicular to the PSBs (Figures 1C and 1E). Moreover, this bias is the same on either side of the boundary (either *wingless* or *engrailed*-expressing cells) (Figures S1C and S1D).

Hertwig’s rule predicts that the plane of cell division is perpendicular to the long axis of the interphase cell (di Pietro et al., 2016, Hertwig, 1884, Minc et al., 2011). To ascertain if cells were following this long axis rule, we imaged dividing cells in a live embryo, using E-cadherin-GFP to monitor cell shapes, and the microtubule binding protein Jupiter-Cherry (Bergstralh et al., 2015) to visualize the mitotic spindle (Figure 1F). We found that the metaphase spindle moves dynamically, stabilizing at anaphase onset (see Figures 5A–5C). For each cell division, we determined the principal axis of cell shape prior to nuclear envelope breakdown (NEBD) and compared its orientation with the mitotic spindle orientation at anaphase (Figures 1F–1J). We discovered that 75% of cells at NEBD have their long axis oriented perpendicular to the AP embryonic axis, and this is true of both BCs and NBCs (Figure S1F). However, while NBCs follow the long axis rule, with a strong correlation between angle of cell division and angle of cell shape relative to AP (*r* = 0.48; Figure 1G), BCs do not (*r* = 0.17; Figures 1H and 1I). This could have been explained by the finding that BCs are less strongly elongated than NBCs (Figure S1G). However, we found that while highly elongated BCs divide according to their shapes, the moderately elongated BCs do not follow the long axis rule, in contrast with the NBCs of equivalent aspect ratio (Figures 1J, S1E, and S1H). These results suggest that additional cue(s), other than cell shape, control the orientation of BC divisions.

### Actomyosin Cables Are Necessary and Sufficient to Orient Boundary Cell Divisions

**Figure 2:**
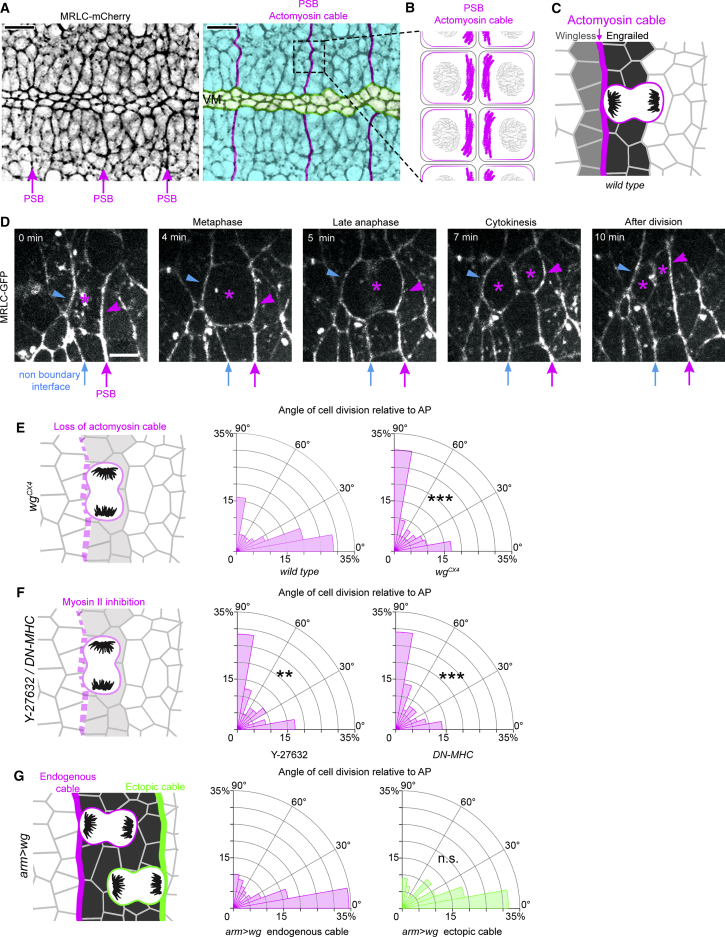
The Actomyosin Cable at the PSB Is Necessary and Sufficient to Orient Boundary Cell Division (A) Image from an MRLC-Cherry movie. Arrows label actomyosin cables at PSBs. Color-coded version of the same image: PSB actomyosin cables highlighted in magenta, extended germband tissue in cyan, ventral midline (VM) in green. (B) Diagram representing a zoomed-in PSB actomyosin cable. The cable is formed by apposed actomyosin-enriched cell cortices on either side (Monier et al., 2010). (C) Diagram representing AP-oriented cell divisions at the PSB. (D) The actomyosin cable at the PSB is maintained throughout cell division. Time-lapse images of a dividing cell in an MRLC-GFP-expressing embryo. Asterisk indicates the dividing cell and its daughters. Magenta arrow: PSB, blue arrow: non-boundary interface. Scale bar, 5 μm. (E) Cell division angles relative to AP in WT embryos and *wg*^*CX4*^ mutants (both quantifications for stage 9 to 11 embryos) (WT, n = 418; *wg*^*CX4*^, n = 378; Mann-Whitney test, *U* = 58262, ^∗∗∗^p < 0.0001). (F) Cell division angles relative to AP in embryos injected with the Rok inhibitor Y-27632 (n = 81; Mann-Whitney test, *U* = 2548, ^∗∗^p = 0.0073) or expressing DN-MHC (n = 238; Mann-Whitney test, *U* = 23586, ^∗∗∗^p < 0.0001) to inhibit Myosin II activity. (G) Cell division angles relative to AP in embryos overexpressing Wingless (*arm > wg*) to generate an ectopic actomyosin cable (green). Ectopic boundary cells contacting the ectopic actomyosin cable (right, n = 133) orient their divisions similarly to the boundary cells at endogenous PSB (left, n = 218; Mann-Whitney test, *U* = 14489, p = 0.994).

PSB cell-cell interfaces are enriched in actomyosin and form tissue-scale contractile cables (Figures 2A and 2B) that act as mechanical barriers limiting cell mixing caused by cell divisions and, earlier in development, polarized cell intercalations (Monier et al., 2010, Tetley et al., 2016). Since force anisotropies have been reported to control cell division orientation *in vitro* (Fink et al., 2011) as well as in tissues (Campinho et al., 2013, Mao et al., 2013, Wyatt et al., 2015), we hypothesized that the actomyosin cable at PSBs might act as a source of anisotropic tension during mitosis. As previously reported (Monier et al., 2010), live imaging using GFP-tagged Myosin II Regulatory Light Chain (MRLC-GFP) and quantification of fluorescence intensity at boundary versus non-boundary interfaces of dividing cells showed that the actomyosin cable-like enrichment persists at the cortex of boundary cells during division (Figures 2C, 2D, and S2A). We asked whether the actomyosin cable is required for the division orientation bias we observed in these cells. We examined *wingless (wg)* null mutant embryos, where actomyosin fails to accumulate at PSBs (Monier et al., 2010, Tetley et al., 2016, Urbano et al., 2018) (Figure 2E). Strikingly, the majority of BCs now divide perpendicular to AP like NBCs (Figures 2E, S2B, and S2C). To test if this difference was caused by the loss of actomyosin enrichment in *wg* mutants, we inhibited Myosin II activity in two different ways. First, we injected wild-type embryos with a concentration of the Rok inhibitor Y-27632 that does not affect cell division but does disrupt boundary function (Monier et al., 2010, Urbano et al., 2018). Second, we overexpressed a dominant-negative form of the Myosin II Heavy Chain in the epidermis (Franke et al., 2005, Monier et al., 2010). Both experiments disrupt the division orientation bias in BCs as in *wg* mutants (Figures 2F and S2D–S2G). These experiments indicate that the actomyosin cable at PSBs is required for orienting the BCs’ divisions perpendicular to the boundary. Next, we asked whether BCs follow the long axis rule when actomyosin contractility is inhibited. We live-imaged embryos injected with Y-27632 and examined cell shape orientation prior to division, as before. This analysis showed that, indeed, BCs follow the long axis rule in Y-27632 but not H_2_O-injected embryos (Figures S2I–S2K). We further checked that Y-27632 treatment did not affect cell shape orientation or elongation (Figures S2L and S2M). These results indicate that in absence of a contractile actomyosin cable at the boundary, BCs behave as NBCs.

Next, to ascertain if a cable-like actomyosin enrichment is sufficient to orient cell division, we generated an ectopic cable at the posterior interface of the engrailed expression domain by uniformly expressing Wg in the epidermis (Urbano et al., 2018) (Figure 2G). We have shown previously that this interface behaves as an ectopic PSB, with a similar actomyosin enrichment and increased interfacial tension as for endogenous PSBs (Urbano et al., 2018). We found that cells contacting this ectopic cable, orient their division perpendicular to it, as at the endogenous cable (Figures 2G and S2H). Taken together, the above findings indicate that a contractile actomyosin cable is both necessary and sufficient to orient boundary cell division.

### Elevated Tension at PSB Actomyosin Cables Is Required for Orienting Cell Division

Since PSB actomyosin cables act as mechanical barriers preventing cell mixing during body axis elongation (Tetley et al., 2016) and segmentation (Monier et al., 2010), we postulated they might orient cell divisions as a consequence of their mechanical properties. Cortical tension can be estimated by severing cell-cell junctions and comparing recoil velocities, assuming that friction is the same for all junctions (Farhadifar et al., 2007, Rauzi and Lenne, 2015). Using this approach, we have shown previously and confirm here that cortical tension at PSB junctional interfaces is elevated, about 2-fold that of non-PSB interfaces (Figures S3A–S3F) (Tetley et al., 2016, Urbano et al., 2018). Based on these measurements, we hypothesized that cells at the boundary might experience a cortical anisotropy in tension that biases their division orientation (Fink et al., 2011).

**Figure 3:**
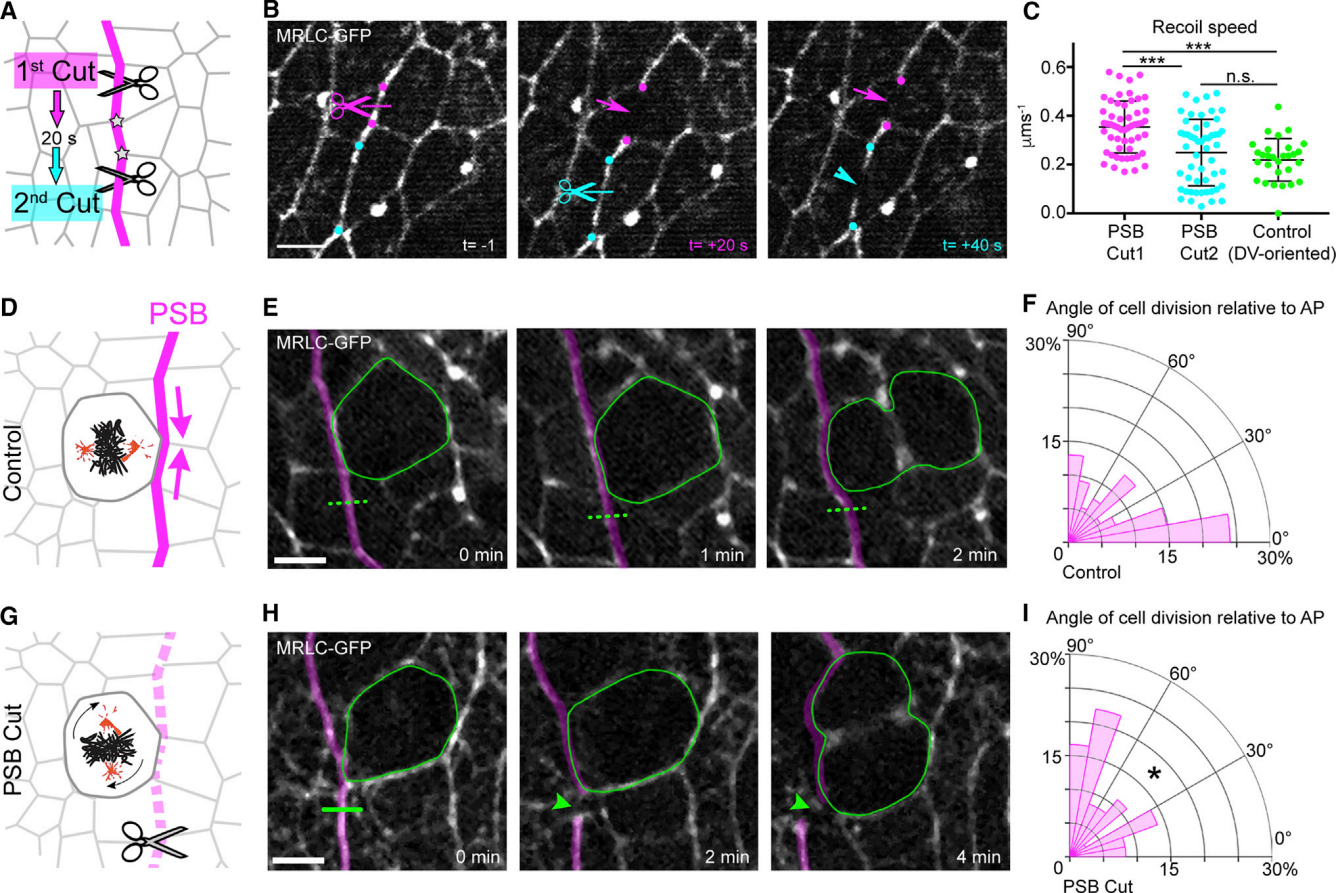
Anisotropic Local Tension at the Actomyosin Cable Is Required for Cell Division Orientation of Boundary Cells (A) Diagram representing a consecutive ablation experiment. The actomyosin cable is cut once and allowed to relax. After 20 s, when relaxation is maximal, a second cut is performed at a distance of two cell vertices away from the first cut (stars). (B) Still images from a consecutive ablation experiment. The site of the first cut and its recoiling cut ends are highlighted in magenta, while the second cut site is highlighted in cyan. Scale bar, 5 μm. (C) Speed of recoil upon ablation at first and second sites at PSB and at non-PSB DV-oriented junctions as a control (Cut1, n = 52; Cut2, n = 52; Control DV, n = 29; Kruskal-Wallis tests; Cut1 versus Cut2, *H* = 26.44, ^∗∗∗^p = 0.0002; Cut1 versus Control, *H* = 26.44, ^∗∗∗^p < 0.0001; Cut2 versus Control, *H* = 26.44, p = 0.573). Means ± SDs shown. To impair tension locally at the PSB, the actomyosin cable is either treated with control low-intensity laser light (D) or cut (G) next to a mitotic boundary cell in metaphase and the orientation of the cell division is measured. Still images following a cell division during a loss of PSB tension experiment (H) and its control (E). Small regions (green lines) of the PSB actomyosin cables (magenta) were illuminated using the laser settings (25% power) for image acquisition as a control (E) or cut using the laser at 100% power (H). Green arrowhead highlights cable recoil. Scale bar, 5 μm. (F) Cell division angles relative to AP for the control and (I) PSB-ablated cells (Control, n = 54; PSB cuts, n = 36; Mann-Whitney test, *U* = 717, ^∗^p = 0.0356).

We developed a strategy to decrease tension locally at the PSB, in order to investigate whether this would change the division orientation of boundary cells in contact with the cable. We reasoned that a single cut in the actomyosin cable could decrease tension along some of its length. To check if this assumption was correct, we ablated the cable twice, leaving an interval of 20 s before the second cut was performed one cell diameter away further along the cable (Figures 3A and 3B) (Rudolf et al., 2015). Comparison of recoil velocities at first and second ablation sites show a nearly 2-fold decrease in recoil speed, indicating that a single cut effectively reduces tension in the cable (Figures 3C and S3G–S3I). This is consistent with contractile stress being propagated and integrated along actomyosin-enriched supracellular cables (Fernandez-Gonzalez et al., 2009). Based on this finding, we cut the PSB actomyosin cable in proximity of boundary cells at metaphase, when the spindle is moving dynamically (see Figures 5A–5C), to check whether a tensile cable was required for their division orientation. To efficiently ablate the PSB cable for the duration of the division, we repeated the laser ablation every 25 s to suppress actomyosin-mediated wound healing (Fernandez-Gonzalez and Zallen, 2013). Kymographs were inspected to check that no repair occurred and that the cable had lost tension throughout the experiment (Figure S3J). As a control, we targeted the cable near metaphase cells using low-intensity laser light (Figure 3D). We found that loss of tension at the actomyosin cable significantly reduced the ability of BCs to orient their divisions perpendicular to the cable, compared to control cells (Figures 3D–3I), despite their cell shapes being indistinguishable from those of control BCs (Figures S3K and S3L). Note that upon ablation, we found only a marginal decrease in Myosin II along the cable (about 10%, Figure S3I, see also Discussion), suggesting that it is the loss of tension, rather than a loss of actomyosin enrichment, which is the important cue. In summary, these experiments indicate that a cortical anisotropy of tension at the PSB is necessary to orient boundary cell divisions.

### Boundary Cells Do Not Require Pins, Mud, or the Vertex Rule to Orient Their Divisions

In mammalian cells, E-cadherin recruits the cortical regulator LGN (Pins in *Drosophila*) (Izumi et al., 2006) to adherens junctions (Gloerich et al., 2017). When epithelial monolayers are stretched, LGN/Pins is required to align cell division orientation with the force direction, independently of cell shape (Hart et al., 2017). We therefore investigated Pins requirement for the cell division orientation bias at PSBs. Knockdown of Pins, either by RNAi (Figures S4D–S4F) or by removing both maternal and zygotic Pins with *pins*^*p62*^ germline clones, causes a substantial fraction of the divisions to happen out of the tissue plane (Figures S4G and S4J). This is expected since Pins is required for spindle orientation perpendicular to the tissue apico-basal axis (Izumi et al., 2006). Of the remaining divisions occurring within the tissue plane, we did not find a difference in cell division orientation for either BCs or NBCs (Figures S4H, S4I, S4K, and S4L). These results suggest that Pins is not required to orient BC divisions, relative to the PSB.

**Figure 4:**
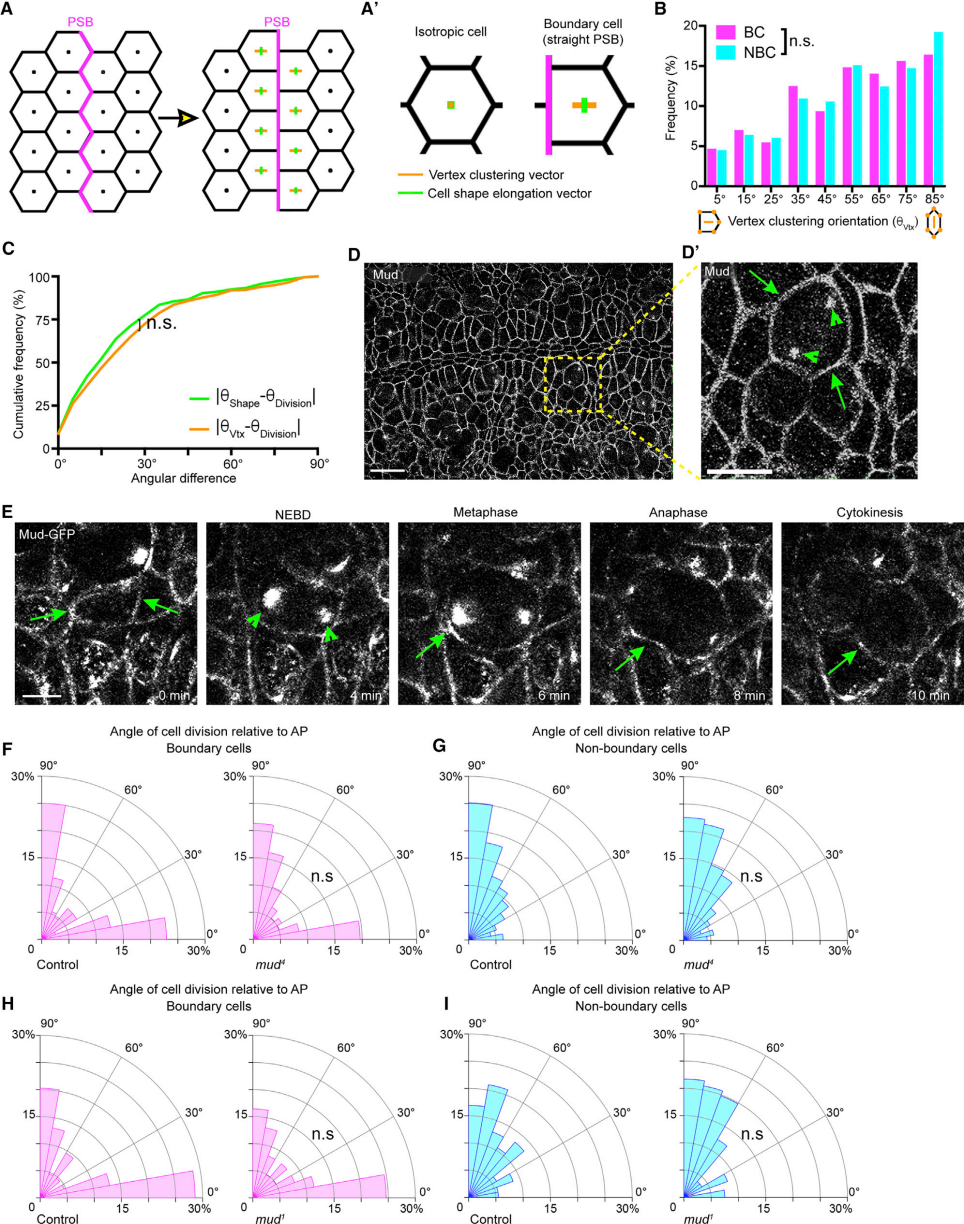
Boundary Cells Do Not Require the Vertex Rule to Orient Their Divisions (A) For a given hexagonal starting configuration of cells (left panel), imposing a straight boundary (PSB, thick magenta line, right panel) introduces both clustering of tricellular vertices perpendicular to the boundary and a small cell shape elongation parallel to it. (Aʹ, inset of A) While for an isotropic hexagonal cell the vectors η_Vertex_ (orange) and η_Shape_ (green) equal 0, for cells with a straight boundary, they diverge in their orientation and magnitude, with η_Vertex_ perpendicular to the boundary (with a magnitude of 0.21) and η_Shape_ parallel to it (magnitude of 0.13). Thus, a straight multicellular interface could in itself introduce a cell division orientation bias if the orientation mechanism depended on the clustering of cell vertices. (B) Histogram of vertex cluster orientation in relation to the AP axis of the embryo for BC and NBC at t = −12 min from cytokinesis from E-cadherin-GFP, MRLC-mCherry movies (n = 265, NBC; n = 128, BC; Mann-Whitney test, *U* = 16,477, p = 0.647). (C) Cumulative histogram of the shape and vertex angular differences for all tracked cell divisions (n = 359 from 5 embryos; Kolmogorov-Smirnov test, *D* = 0.083, p = 0.163). (D) Immunostaining of a stage 9 embryo with anti-Mud antibody. Scale bar, 20 μm. (Dʹ) Inset of D. Arrowheads, endogenous Mud localization at mitotic spindle poles; arrows, localization of Mud at bicellular junctions. Scale bar, 10 μm. (E) Stills of a dividing cell from a Mud-GFP-expressing embryo. Arrowheads, endogenous Mud localization at mitotic spindle poles; arrows, localization of Mud at bicellular junctions. Scale bar, 5 μm. (F) Angles of cell division orientation relative to AP for boundary cells (BC) in *mud*^*4*^ (n = 117), or Control embryos (n = 148; Mann-Whitney test, *U* = 8,215, p = 0.475). (G) Histogram of cell division orientation for non-boundary cells in *mud*^*4*^ (n = 231), or Control embryos (n = 314; Mann-Whitney test, *U* = 35281, p= 0.587). (H) Angles of cell division orientation relative to AP for BC in *mud*^*1*^ (n = 61), or Control embryos (n = 84; Mann-Whitney test, *U* = 2,471, p = 0.717). (I) Angles of cell division orientation relative to AP for NBC in *mud*^*1*^ (n = 106), or Control (n = 219; Mann-Whitney test, *U* = 10,333, p = 0.109).

In the *Drosophila* notum, cells align their divisions according to the spatial distribution of their tricellular vertices (Bosveld et al., 2016). Tissue forces influence these vertex positions, and in turn vertices orient mitoses by anchoring astral microtubules to the cell cortex via Mud, the NuMA homolog (Bosveld et al., 2016). In the notum, the direction of the vector resulting from the spatial distribution of the tricellular vertices of a cell (vertex distribution) is a better predictor of the orientation of division than cell elongation (Bosveld et al., 2016). Because they are tensile structures, PSBs are straighter than non-boundary columns of cell contacts (Monier et al., 2010, Tetley et al., 2016). In theory, this straightness of cell contacts could cluster vertices along the PSB, changing their spatial distribution, which could, in turn, explain the orientation of BC divisions (Figures 4A and 4A′). To test this, we imaged live embryos expressing E-cadherin-GFP (Huang et al., 2009) and MRLC-mCherry (Martin et al., 2009) and tracked 359 dividing cells (See STAR Methods). We found that the tracked BCs do not have a distribution of vertices significantly changed compared to NBCs (Figure 4B). Moreover, the vertex distribution of boundary cells is not better than their elongation at predicting the cell division orientation bias toward the PSB (Figures 4C and S4M–S4O).

Consistent with this result, we find that Mud is not localized at vertices in the embryo (Figures 4D and S4E). In contrast to Pins, however, Mud is mildly enriched at PSBs (Figures S4A–S4C), so we tested the requirement for Mud by analyzing *mud* zygotic mutant embryos, using two different loss-of-function alleles, mud^*4*^ (Ségalen et al., 2010) and mud^*1*^ (Izumi et al., 2006), in which endogenous Mud is not detected (Figure S4P; Izumi et al., 2006). Mud controls epithelial cell division orientation in the plane of the epithelium (Bosveld et al., 2016, Izumi et al., 2006), and indeed we found that 45% of cell divisions occurred out of plane (Figures S4Q and S4R). Restricting our analysis to the remaining planar mitoses, we found that Mud loss of function did not change cell division orientation for either BCs or NBCs (Figures 4F–4I). Together, these results show that the planar division orientation of boundary cells is neither dependent on Pins/LGN nor does it rely on tricellular vertex distribution of Mud/NuMA.

### PSB Actomyosin Enrichment Restricts Spindle Pole Motility

**Figure 5:**
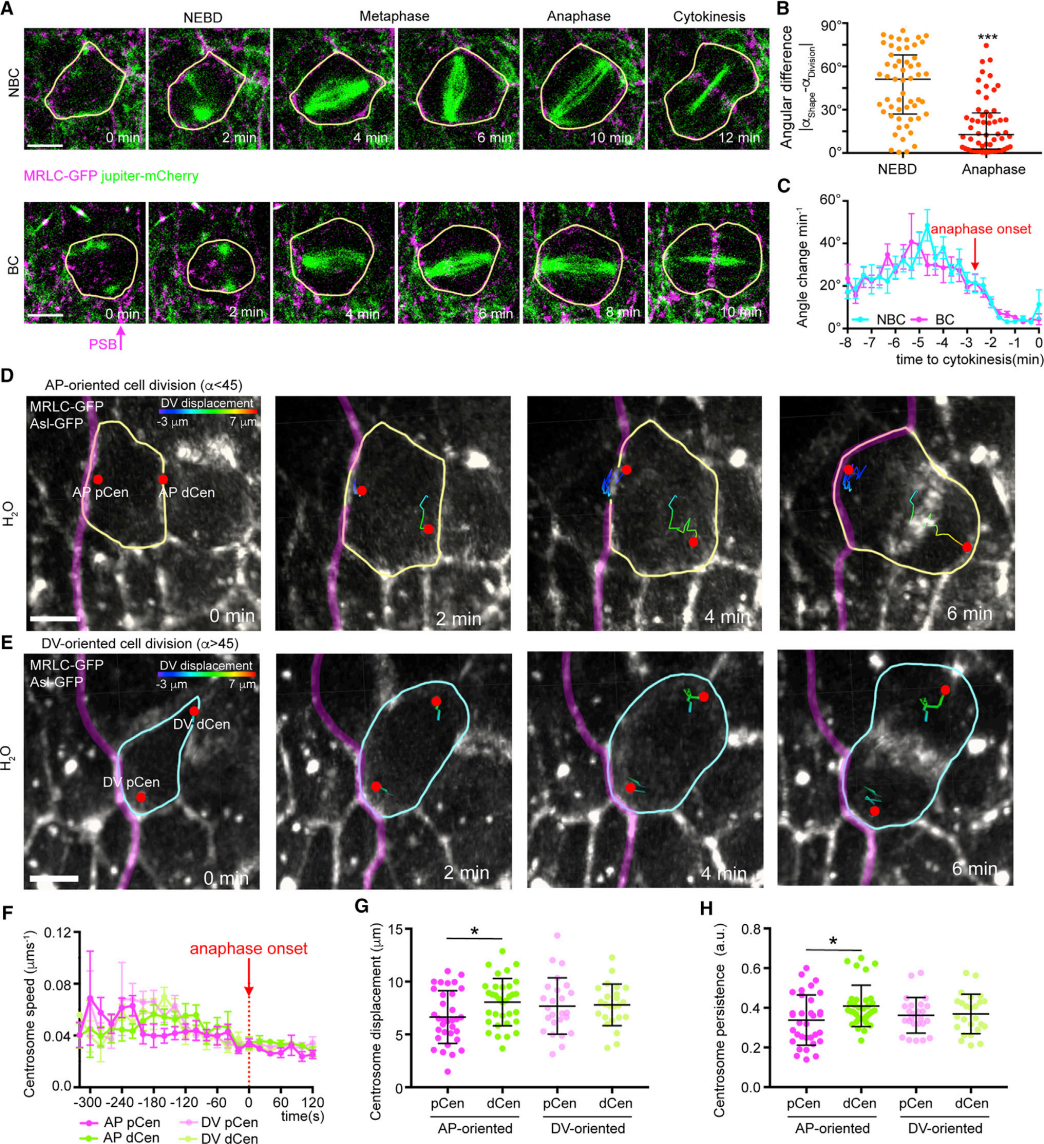
Spindle Pole Motility Is Restricted by the PSB (A) Still images from a time-lapse movie of an MRLC-GFP and Jupiter-Cherry-expressing embryo. A representative non-boundary cell (top) and boundary cell are shown. Scale bar, 5 μm. (B) Angular difference between shape and cell division at NEBD and anaphase (n = 56; Kolmogorov-Smirnov test, *D* = 0.532, ^∗∗∗^p < 0.001). Median ± interquartile range are shown. (C) Angular rotation per minute of the mitotic spindle for BC (n = 27) and NBC (n = 28) from NEBD to cytokinesis. t = 0, cytokinesis. Mean ± SEM are shown. Representative AP-oriented (D) (α < 45) and DV-oriented (E) (α > 45) BC cell division from an H_2_O injected embryo expressing MRLC-GFP and Asl-GFP. Centrosome tracks are highlighted and color-coded for DV displacement (see Figures S5B and S5C). pCen and dCen, centrosomes proximal and distal from PSB, respectively. Scale bar, 5 μm. (F) Centrosome speed from NEBD to cytokinesis (AP pCen, n = 16; AP dCen, n = 16; DV pCen, n = 19; DV dCen, n = 19). t = 0, anaphase onset. Mean ± SEM. are shown. (G) Total displacement for each centrosome from NEBD to cytokinesis (AP pCen, n = 33; AP dCen, n = 33; DV pCen, n = 25; DV dCen, n = 25; one-way ANOVA, *F* = 2.252, p = 0.059; AP pCen versus AP dCen, ^∗^p = 0.0317; all other comparisons p > 0.30). Means ± SDs are shown. (H) Persistence for each centrosome from NEBD to cytokinesis (AP pCen, n = 33; AP dCen, n = 33; DV pCen, n = 25; DV dCen, n = 25; one-way ANOVA, *F* = 2.863, p = 0.040; AP pCen versus AP dCen, ^∗^p = 0.0241; all other comparisons p > 0.20). Means ± SDs are shown.

An alternative explanation for the oriented division in BCs is that Myosin-mediated contractility at the PSB directly impacts spindle pole positioning, especially as Myosin-II is required for spindle pole separation, both in mammalian cells (Rosenblatt et al., 2004) and in *C. elegans* embryos (De Simone et al., 2016). To explore this, we characterized the dynamics of spindle pole motility in BCs versus NBCs. Imaging of mitotic spindles using Jupiter-Cherry revealed a rotational behavior from NEBD throughout metaphase in both NBCs and BCs, which decreases during the 2 min before anaphase onset, when the spindle is stabilized (Figures 5A–5C). As in vertebrate embryos (Larson and Bement, 2017), the orientation of the mitotic spindle poorly correlated with cell shape at NEBD, and this correlation significantly improved at anaphase, indicating that mitotic spindles adopt their final orientation just before anaphase (Figure 5B). We did not, however, observe any differences in spindle dynamics between BCs and NBCs (Figure 5C).

Next, we investigated whether the mitotic spindle pole closest to the PSB behaved differently from the pole more distal from the PSB. To separately track spindle poles, we imaged embryos expressing the centrosome marker Asl-GFP (Blachon et al., 2008) and MRLC-GFP to identify the PSB. Tracking spindle poles from NEBD to cytokinesis (Figures 5D and 5E) showed that the speed and the distance covered by poles throughout mitosis is comparable (Figures 5F and S5A). However, in BCs dividing perpendicular to the boundary (AP-oriented, Figure 5D), the centrosome proximal to the PSB (pCen) was displaced less (Figure 5G), especially along the dorsoventral (DV) direction (Figure S5B). Also, proximal centrosomes displayed a trajectory significantly less persistent (we define persistence as the ratio between centrosome displacement and the total distance covered) than centrosomes distal from the PSB (dCen) (Figure 5H). In contrast, proximal and distal centrosomes of BCs dividing without a bias toward the boundary (DV-oriented, Figure 5E), behave similarly (Figures 5G, 5H, and S5C). Moreover, in Y-27632 treated embryos, each centrosome displays comparable motility even in BCs that divide perpendicular to the PSB (Figures S5D–S5I). This suggests that the PSB, through the action of Myosin II, orientates BC cell division by capturing the more proximal centrosome and limiting its motility.

### Ectopic Tension Anisotropy Is Sufficient to Orient Cell Divisions

**Figure 6:**
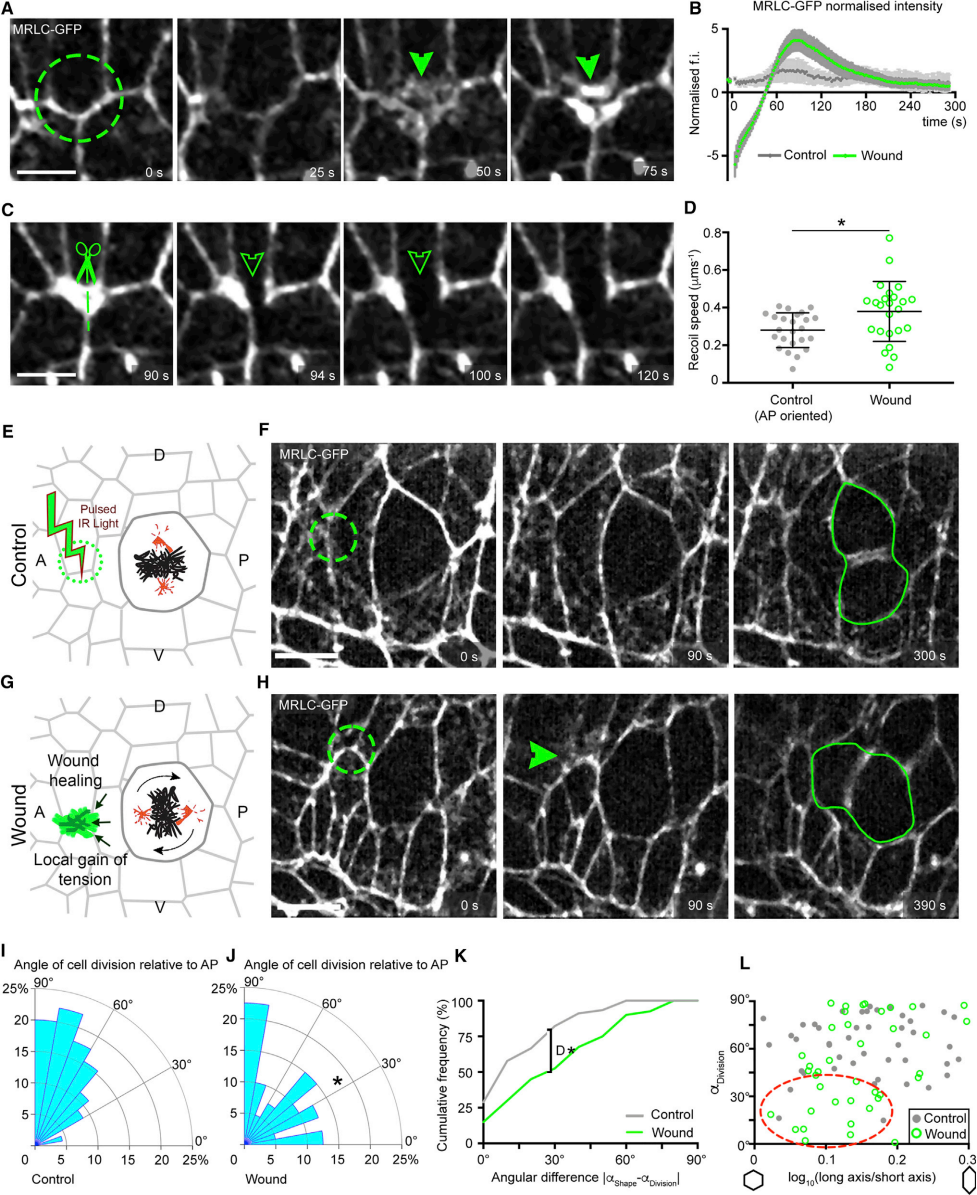
Anisotropic Tension Generated Locally by Laser Wounding Is Sufficient to Orient the Division of Non-boundary Cells (A) Laser irradiation of a small region of the epidermis (green circle) results in a wound healing response with transient accumulation of a medial MRLC-GFP meshwork. Scale bar, 5 μm. (B) Normalized fluorescence intensity of the irradiated area over time (n = 26, wound; n = 19, control not irradiated). Means ± SEM are shown. Myosin intensity peaks at t = 90 s after irradiation. (C) Laser ablation of the Myosin II meshwork displays rapid recoil. Scale bar, 5 μm. (D) Quantification of the recoil speed of the ablated Myosin II meshwork, compared to ablation of AP-oriented junctions in unwounded tissue (n = 24, wound; n = 24, control; Student’s t test, *t* = 2.65, ^∗^p = 0.016). Means ± SDs are shown. To increase tension locally, a wound is performed by laser irradiation of a small region (green circles) on the anterior or posterior side of a mitotic non-boundary cell in metaphase, at a distance of about one cell diameter away. The irradiation is either the same as for image acquisition (25% laser power) as a control (E) or at 80% power for ablation (G). Still images of an example experiment are shown for a control (F) and a wounding assay (H). Green arrowhead highlights myosin accumulation upon wounding. Scale bar, 10 μm. Angles of cell division relative to AP for control (I) and wounded cells (J) (n = 45, control; n = 40, wound; one-tailed Mann-Whitney test, *U* = 682, ^∗^p = 0.027). (K). Cumulative frequency distribution of the difference between the orientation of cell division and the orientation of the principal axis of cell shape for NBC near a wound (green) and control NBC (gray) (n = 45, control; n = 40, wound; Kolmogorov-Smirnov test, *D* = 0.3278, ^∗^p = 0.0211). (L) Angles of cell division as a function of cell elongation, measured as log_10_(long axis/short axis). Red circle highlights NBC cells (green) moderately elongated which are dividing along AP (α < 45°) upon wounding, (log_10_(long axis/short axis) < 0.2) (n = 45, Control; n = 40, Wound; one-tailed Mann-Whitney test, *U* = 682, p = 0.027).

Our results so far suggest that tension anisotropy caused by actomyosin cables can orient cell division via a centrosome capture mechanism. To test whether tension anisotropy is sufficient to orient mitoses *in vivo*, we sought to change tension in the vicinity of NBCs, by laser wounding the nearby epithelium to provoke a wound healing response (Campinho et al., 2013, Fernandez-Gonzalez and Zallen, 2013). We found that apical Myosin II accumulates fast at small wounds, peaking at about 90 s after wounding (Figures 6A and 6B). To ascertain whether this enrichment increases cortical tension, we ablated the Myosin-II meshwork recruited in the repairing region and measured recoil speed (Figure 6C). The ablated Myosin-II meshwork recoils significantly faster than ablated control junctions, suggesting it is under greater tension (Figure 6D). Thus, using a treatment that does not have any detectable detrimental effects on neighboring cells (for example, we do not observe any cell delamination, Figures S6A and S6B), we can locally increase cortical tension with good spatial and temporal resolution.

With this new tool in hand, we sought to locally increase tension at the vicinity of a metaphase NBC, either on its anterior or posterior side (Figures 6G and 6H). Controls were performed by irradiating a region of the same size with low laser light (Figures 6E and 6F). Whereas control cells divide mostly along DV as expected (Figures 6F and 6I), we found that, in contrast, NBCs next to an A or P source of tension divide more frequently along the AP axis (Figures 6H and 6J). We checked whether this is caused by a change in cell geometry (Figures S6C and S6D). NBCs near a wound no longer followed the long axis rule as well as control cells (Figure 6K); with this effect being more pronounced for moderately elongated cells (red oval, Figure 6L). We conclude that a local anisotropy in tension mediated by Myosin II is sufficient to orient cell division *in vivo*.

## Discussion

Our study demonstrates that *in vivo*, dividing cells can orient their plane of division in response to a local anisotropy in actomyosin-mediated cortical tension. This overrides the influence of cell geometry, if cells are not too elongated. This finding adds an important insight to *in vivo* studies of cell division where it is unclear if forces act directly, or indirectly via a change in cell geometry, because tissue-scale forces elongate cells (Campinho et al., 2013, Mao et al., 2011, Mao et al., 2013).

We found that in all the experimental conditions where cells have one side of the cortex belonging to a tensile actomyosin-enriched parasegmental boundary, their division orientation is biased by the boundary rather than by the cells’ longest axis. This is true unless the boundary cells are very elongated (Figure 1J), in which case the mechanical boundary is unable to reorient the dividing cells. We observed the same result in our wounding assay to generate high tension locally: the cells that are not reoriented are the most elongated (Figure 6L). This suggests that above a certain threshold of cell elongation, which we estimate being around 2 for the ratio between long axis and short axis (Figures 1J and S1H), the long axis rule “wins” over localized cortical actomyosin tension. This is likely to explain the bimodal distribution of division orientation we observe in our fixed data: although a high proportion of BCs divide perpendicular to the boundary (along AP), there is always a group of cells that still divide along DV (Figure 1E). Imaging live embryos, we show these are indeed the strongly elongated cells (Figure S1H). We also found that when tension is disrupted at the PSBs, the boundary cells now divide according to the orientation of their long axis, which is on average along DV (Figures 3I and S3K). This suggests that the default orientation cue in this epithelium is the cell’s shape. It is still unclear why cells tend to divide according to their long axis. For strongly elongated cells, it is possible these do not manage to round up completely and that steric hindrance of the spindle constrains its orientation (Lázaro-Diéguez et al., 2015, Williams et al., 2011). For moderately elongated cells, such an explanation is unlikely. It was recently discovered that the spatial distribution of vertices in the *Drosophila* notum epithelium predicts the orientation of the division better than the long axis rule, in particular for moderately elongated cells (Bosveld et al., 2016). The explanation for this is that Mud (the homolog of NuMA) interacts with septate junction (similar to tight junctions) components at vertices, causing a redistribution of forces exerted by the astral microtubules. We have ruled out a role for Mud in experiments in this paper, suggesting that the default orientation of cell division in the *Drosophila* embryo epithelium is determined by other mechanisms. Direct force sensing could be involved, although we cannot disentangle this from the long axis rule in NBCs.

In BCs, our results point toward a direct role of actomyosin-mediated tension anisotropy in biasing spindle orientation. We found that the *Drosophila* homolog of LGN, Pins, is not required for the planar orientation bias in BCs (or NBCs). Consistent with this, Myosin II activity was shown not to affect Pins localization in *Drosophila* embryos (Chanet et al., 2017). Since Mud is also not required, this suggests that the Pins/Mud/Dynein complex does not play a role in the planar orientation of the cells in our study (Note that Pins and Mud are, however, required for the division parallel to the plane of the epithelium, see Figures S4G, S4J, S4Q, and S4R; Bosveld et al., 2016, Izumi et al., 2006, Izumi et al., 2004). It is therefore possible that a mechanosensitive pathway in the cells is what responds to tension and interacts with the astral microtubules to orient the spindle. Such a molecular cue could be E-cadherin (den Elzen et al., 2009), whose localization can change when tension at the cortex is decreased or increased (Lecuit and Yap, 2015). Illustrating this possibility, E-cadherin/LGN interactions control cell division orientation in response to tissue stretch in mammalian cells (Hart et al., 2017). Another component of adherens junctions, the protein Canoe/Afadin, is required to attach the actomyosin network to the cortex in early *Drosophil*a embryos (Sawyer et al., 2011). Canoe promotes spindle orientation by recruiting cortical actin via RhoA and the formin Dia in *Drosophila* sensory organ precursors (Johnston et al., 2013). Relevant to this, one of its zebrafish homologs, ZDia2, is required for spindle orientation toward a cortical enrichment of actin during epiboly (Castanon et al., 2013). Future work could test if tension-dependent localization of adherens junctions components such as Canoe or E-cadherin is important for both force integration and spindle orientation at the PSB.

We have found that the centrosome proximal to the actomyosin cable is significantly less mobile during metaphase, suggesting it might be captured by the cortex experiencing high tension. Supporting this idea, cortical actomyosin contractility has been recently shown to promote clustering of centrosomes by limiting their motility in cultured cancer cells with supernumerary centrosomes (Rhys et al., 2018). Forces can capture the mitotic spindle poles via subcortical actin clouds (Fink et al., 2011) or the actin-microtubule-binding motor Myosin10 (Kwon et al., 2015). So far, actin clouds have only been reported in cultured cells (Fink et al., 2011, Kwon et al., 2015) and in *Xenopus* epithelia (Woolner et al., 2008), and Myosin10 is not expressed during early *Drosophila* embryogenesis (Gramates et al., 2017, Lye et al., 2014). Alternatively, actomyosin-mediated tension at the PSBs might cause an anisotropy in cortex stiffness, which could bias the balance of forces orienting the spindle. Indeed, Myosin II, together with the actin-membrane crosslinker Moesin, is essential for cortex stiffening during mitotic cell rounding (Chanet et al., 2017, Kunda et al., 2008). Moreover, impairment of cortical stiffening by actin-depolymerizing drugs or Myosin II inhibition perturbs cell division orientation *in vivo* (Chanet et al., 2017, Luxenburg et al., 2011, Nakajima et al., 2013). A stiff enough actomyosin cortex is essential to balance the tension that cortical force generators exert when pulling on the spindle in *C. elegans* (Redemann et al., 2010) and to drive asymmetric spindle localization in mouse oocytes (Chaigne et al., 2015).

Our study uncovers an effect of actomyosin supracellular cables in orienting cell divisions *in vivo*. Actomyosin cables are very common in developing epithelia and are found not only at compartmental boundaries but also during tissue closure, wound healing, tube formation, and convergence extension of tissues (Röper, 2013). In the wing disc, where actomyosin supracellular tension has been identified at both AP and DV boundaries, no orientation of cell division perpendicular to the boundary has been reported (Aliee et al., 2012, Landsberg et al., 2009, Umetsu et al., 2014). However, the tension at the AP boundary is controlled cell-autonomously by Hedgehog signaling (Rudolf et al., 2015). In the embryo, in contrast, we found that severing the actomyosin cable causes tension to be lost non-cell-autonomously along the cable. We also found that Myosin II is decreased along the cable as a consequence of this loss of tension (Figure S3I). This indicates that in the embryo, actomyosin cables are supracellular tensile structures, where Myosin II enrichment might be reinforced by a mechanosensitive feedback (Fernandez-Gonzalez et al., 2009). In contrast with the wing disc, the embryonic ectoderm is a simple epithelium lacking septate junction or extracellular matrix, where force transmission along an actomyosin cable might be facilitated. This might, in turn, influence how much tension is generated and how much it can bias division orientation.

What function may tension-generated orientation of cell division serve during morphogenesis? In the tissue studied here, cell divisions start toward the end of axis extension (Martinez-Arias, 1993), when large-scale polarized cell intercalations have generated a disordered epithelium (Zallen and Zallen, 2004). It is possible that this intense period of cell division is required to restore optimal tissue packing (Gibson et al., 2006, Gibson et al., 2011). In this context, the separation of metameric units by mechanical barriers that bias cell division in a tension-dependent manner might be key for the conservation of shape between parasegments. More generally, our study raises the possibility that cell division orientation biases directly caused by local tension anisotropies could be important in the maintenance of correct tissue and organ sizes during growth.

## STAR★Methods

### Key Resources Table

**Table:** 

REAGENT or RESOURCE	SOURCE	IDENTIFIER
**Antibodies**		

Rabbit Phospho-histone H3	Cell Signaling	Cat #9701; RRID:AB_331535
Rabbit anti-Engrailed	Santa Cruz Biotechnology	Cat # sc-28640; RRID:AB_640146
Goat anti-GFP-FITC	Abcam	Cat #ab6556;
Guinea pig anti-Sqh-1-P	Zhang and Ward (2011)	N/A
Mouse anti-phospho-Tyrosine	Cell Signaling	Cat #9411; RRID:AB_331228
Mouse anti-Wingless	DSHB	Cat #4F3;
Rabbit anti-Pins	Izumi et al. (2006)	N/A
Rabbit anti-Mud	Izumi et al. (2006)	N/A

**Chemicals, Peptides, and Recombinant Proteins**		

Y-27632	TOCRIS	Cat # 1254; CAS:129830-38-2

**Deposited Data**		

Deposited raw datasets on Mendeley Data	This paper	https://doi.org/10.17632/r4tdprzd8v.1

**Experimental Models: Organisms/Strains**		

*Drosophila melanogaster*: y[1]w[67c23] (yw^67^)	Bloomington Drosophila Stock Center	BDSC:6599; RRID:BDSC_6599
*Drosophila melanogaster*: en-lacZ	Busturia and Morata (1988)	FBal0041284
*Drosophila melanogaster*: arm-Gal4	Sanson et al. (1996)	FBal0058766
*Drosophila melanogaster*: UAS-wg	Lawrence et al. (1995)	FBtp0001287
*Drosophila melanogaster*: arm-FRT-stop-FRT Gal4::VP16	Sanson et al. (1996)	FBal0058766
*Drosophila melanogaster*: KB19	Sanson et al. (1996)	FBtp0001121
*Drosophila melanogaster*: UAS-GFP::DN-MHC	Franke et al. (2005)	FBal0190636
*Drosophila melanogaster*: ubi-E-Cadherin::GFP	Oda and Tsukita (2001)	FBal0122908
*Drosophila melanogaster*: Jupiter::mCherry	Bergstralh et al. (2015)	N/A
*Drosophila melanogaster*: en-Venus	Umetsu et al. (2014)	N/A
*Drosophila melanogaster*: osk-Gal4::VP16	Bloomington Drosophila Stock Center	BDSC: 44242
*Drosophila melanogaster*: RNAi of Pins: y[1] sc[^∗^] v[1]; P{TRiP.GL00622}attP40	Bloomington Drosophila Stock Center	BDSC: 37479 FBst0037479
*Drosophila melanogaster*: RNAi of eGFP: y[1] sc[^∗^]v[1];P{y[+t7.7]v[+t1.8]=VALIUM20-EGFP.shRNA.1}attP2	Bloomington Drosophila Stock Center	BDSC: 41556 RRID:BDSC_41556
*Drosophila melanogaster*: Asl-Asl::GFP	Blachon et al. (2008)	N/A
*Drosophila melanogaster*: *GFP-Mud62E1.GFP-Mud65B* (Bosveld et al., 2016)	Bosveld et al. (2016)	FBtp0111861
*Drosophila melanogaster*: wg^CX4^	Baker (1987)	N/A
*Drosophila melanogaster*: mud^4^	Yu et al. (2006)	BDSC: 9563 FBal0012574
*Drosophila melanogaster*: mud^1^	Yu et al. (2006)	BDSC:9562 FBst0009562
*Drosophila melanogaster*: asl^B46^	Baumbach et al. (2015)	N/A
*Drosophila melanogaster*: sqh^AX3^	Jordan and Karess (1997)	FBal0035707
*Drosophila melanogaster*: sqh-sqhGFP42	Royou et al. (2004)	FBal0221190
*Drosophila melanogaster*: GAP43^mem^::mCherry	Martin et al. (2010)	FBal0258719
*Drosophila melanogaster*: shotgun::GFP	Huang et al., (2009)	FBti0168565
*Drosophila melanogaster*: sqh::mCherry	Martin et al. (2009)	FBal0258457
*Drosophila melanogaster*: sqh^AX3^; sqh-sqhGFP42;GAP43^mem^::mCherry/TM6B	Tetley et al. (2016)	N/A
*Drosophila melanogaster*: sqh^AX3^; shotgun::GFP, sqh::mCherry	This paper	N/A
*Drosophila melanogaster*: w[^∗^];P{ry[+t7.2]=neoFRT}82B P{w[+mC]=ovoD1-18}3R/st[1]betaTub85D[D] ss[1] e[s]/TM3,Sb[1]	Bloomington Drosophila Stock Center	BDSC: 2149 RRID: BDSC_2149
*Drosophila melanogaster*: P{ry[+t7.2}=hsFLP}1,y[1]w[1118];Dr[1]/TM3,Sb[1]	Bloomington Drosophila Stock Center	BDSC: 26902 RRID: BDSC_26902
*Drosophila melanogaster*: P{ry[+t7.2]=neoFRT}82B ry[506]	Bloomington Drosophila Stock Center	BDSC: 2035 FBst0002035
*Drosophila melanogaster*: *FRT82B pins*^*p62*^/TM3	Bergstralh et al., 2013, Yu et al., 2000	N/A

**Software and Algorithms**		

Fiji (ImageJ version 2.0.0-rc-68/1.52e	Schneider et al. (2012)	RRID:SCR_002285
GraphPad Prism 7	GraphPad Software Inc.	RRID:SCR_002798
Otracks	Blanchard et al. (2009)	N/A
Matlab 2014a	Mathworks	RRID:SCR_001622
R 3.4.3 GUI 1.70 El Capitan build (7463)	The R Foundation for Statistical Computing http://www.R-project.org	N/A

### Contact for Reagent and Resource Sharing

All information and requests for resources should be directed to the Lead Contact, Bénédicte Sanson (bs251@cam.ac.uk).

### Experimental Model and Subject Details

#### *Drosophila* Strains

*Drosophila melanogaster* were maintained under standard conditions at 25°C. Embryos were collected from stocks or crosses and embryos of both sexes were analysed. All stocks used and their source are listed in the Key Resources Table. In movies, Myosin II Regulatory Light Chain is labelled by *sqh-GFP*; E-Cadherin by *shotgun*::*GFP* or *ubi-E-Cadherin::GFP*; tubulin by *Jupiter::cherry*; cell membranes by *GAP43*::*cherry* and the centrosomes by *Asl-GFP*. In live embryos, the PSBs were identified using *En>Venus* or *sqh-GFP*. To help readers less familiar with *Drosophila* nomenclature, Sqh is indicated throughout the paper as MRLC and Shotgun as E-Cadherin.

#### Genotypes

Figures 1B–1E and S1B–S1D: *yw*^*6*7c23^. Figures 1F–1J and S1E–S1H): *en-Venus/ ubi-E-Cadherin-GFP; Jupiter::mCherry/+*.

Figures 2A and S2A: *sqh*^*AX3*^*,shotgun::GFP;sqh::mCherry*.

(D): *sqh*^*AX3*^*;sqh-sqhGFP42;GAP43*^*mem*^*::mCherry/TM6B.* (E) *yw*^*67*^(left panel), *wg*^*cx4*^*,enLacZ* (right panel). (F) *yw*^*67*^ (left panel), *arm-Gal4::VP16/UAS-DN-MHC::GFP* (right panel). (G) *arm-Gal4/UAS-wg*. The same genotypes were used in Figures S2B–S2H. Figures S2I–S2M: *sqh*^*AX3*^*/+;sqh-sqhGFP42/Asl-Asl::GFP; GAP43*^*mem*^*::mCherry/Asl*^*B46*^.

Figures 3 and S3: *sqh*^*AX3*^*;sqh-sqhGFP42;GAP43*^*mem*^*::mCherry/TM6B*.

Figures 4B, 4C, and S4M–S4O: *sqh*^*AX3*^*; shotgun::GFP; sqh::mCherry*. (D and D’)-Figures S4A–S4C *yw*^*67*^ ^c23^. (E) *GFP-Mud62E1.GFP-Mud65B2/TM6B.* (F and G) and Figures S4P and S4Q *mud*^*4*^/*FTG* or *FTG/y* (described as Control in the figure) or *mud*^*4*^ as indicated. (H and I) and Figure S4R *mud*^*1*^/*FTG* or *FTG/y* (described as Control in the figure) or *mud*^*1*^ as indicated. Figure S4: (D–I) *osk-Gal4::VP16/UAS-eGFP*^*RNAi*^ or *osk-Gal4::VP16/UAS-Pins*^*RNAi*^ as indicated. Figure S4: (J–L) *hsflp70/+;FRT82B/FRT82B* (Control) or hsflp70/+, FRT82BPins^*p62*^*/* FRT82BPins^*p62*^ (MZPins^*p62*^) as indicated.

Figure 5 (A–C)en-Venus/ ubi-DE-Cadherin-GFP; Jupiter::mCherry/+. (D–H)-Figures S5A–S5I *sqh*^*AX3*^*/+;sqh-sqhGFP42/asl-asl::GFP; GAP43*^*mem*^*::mCherry/Asl*^*B46*^.

Figures 6 and S6: *sqh*^*AX3*^; *sqh-sqhGFP42;GAP43*^*mem*^*::mCherry/TM6B*.

#### Germline Clones

*Pins*^*p62*^ mutant embryos analysed in this study are derived from *pins*^*p62*^ germline clones to remove both maternal and zygotic contributions of the gene. Germline clones were generated using the FLP-DFS technique (Chou and Perrimon, 1992). *Pins*^*p62*^*/ovoD1* or *FRT82B/ovoD1* (control) larvae were heat shocked at 37°C for 2 hours for 3 days. Virgins were crossed with *pins*^*p62*^/*TM6c* or *FRT82B/TM6c* (control) males and embryos were analysed.

### Method Details

#### Immunostainings

Embryos collected in a basket from plates containing agar-apple juice were washed in tap water and dechorionated using commercial bleach for 2 minutes, rinsed and dried. Embryos were then fixed at the interface of a 1:1 solution of 37% formaldehyde: 100% heptane for 7 minutes, followed by either manual devitellinisation in PBS 0.1% Triton-X-100 (PTX) or by 100% methanol devitellinisation. Methanol devitellinised embryos were re-hydrated by sequential washes with 75% methanol/PBS, 50% methanol/PBS, 25% methanol/PBS and PBS. They were then blocked in 1% BSA in PTX for 30 minutes and incubated overnight with primary antibodies. Embryos were washed three times in PTX for 5 minutes before secondary antibody incubation for 1 hour at room temperature. Finally, they were washed three more times in PTX and mounted in Vectashield (Vector laboratories) for imaging.

#### Antibodies

The following antibodies were used: Rabbit Phospho-histone H3 (Cell Signalling #9701, 1:200), rabbit anti-Engrailed (Santa Cruz Biotechnology d-300; 1:200), goat anti-GFP-FITC (Abcam ab6556, 1:500), guinea pig anti-Sqh-1P (1:100, (Zhang and Ward, 2011)) (called MRLC-1P in this paper), mouse anti-phospho-Tyrosine (Cell signaling #9411; 1:1000), mouse anti-Wingless (DSHB 4D4; 1:50); mouse anti-Dlg (DSHB 4F3; 1:500) Rabbit anti-Pins (Izumi et al., 2006) (1:1000, a gift from F. Matsuzaki), rabbit anti-Mud(Izumi et al., 2006) (1:200, a gift from F. Matsuzaki). Secondary antibodies conjugated to fluorescent dyes were obtained from Jackson ImmunoResearch Laboratories, Invitrogen and Life Technologies. Cell nuclei were stained using DAPI (Sigma-Aldrich).

#### Confocal Imaging

Embryos were individually mounted in a ventral orientation under a tape bridge on either side of the slide, so that they were sufficiently flattened. Imaging was either performed on a Nikon Eclipse TE2000 microscope coupled to a C1 Plus confocal system (Nikon) and images captured using Nikon EZ-C1 software; or on a Leica TCS SP8 confocal microscope and images captured using LAS X software (Leica). Optical z-stacks were acquired with a depth of 0.5-1 μm between successive optical z-slices. All embryos were imaged using a violet corrected 60x oil objective lens (NA of 1.4). The gain and offset were optimized for each immunostaining and maintained the same between control and mutant conditions.

#### Analysis of Orientation of Cell Division in Fixed Embryos

To analyse cell division orientation in fixed embryos, embryos were immunostained with Phospho-histone H3 antibodies to label mitotic chromosomes, antibodies against Wingless (Wg) or Engrailed (En) to identify the PSB and antibodies against a membrane marker (PTyr or Dlg) to determine cell shapes. Only cells in anaphase or telophase were analysed to ensure that the spindles had finished rotating before fixation. For each cell division, the angle α_PSB_ between the local curvature of the PSB (Figure S1A) and the separating chromosomes was measured using the angle tool in the Fiji software (NIH Image). Angles were always measured as acute angles. To obtain the orientation of cell division in relation to the AP axis, the angle α_PSB_ was transformed as follows: α_division_=90°-α_PSB_, under the assumption that the PSBs are always perpendicular to the AP axis. An example of the α_PSB_ measurement for a BC and a NBC is given in Figures S1B–S1B″.

#### Live Imaging

Dechorionated embryos were transferred into halocarbon oil (Voltalef PCTFE, Arkema), mounted on a stretched oxygen-permeable membrane with their ventral side facing up, and covered by a coverslip, which was supported by a single coverslip bridge on either side of the membrane. Imaging was performed using a Nikon Eclipse E1000 microscope equipped with a spinning disk unit (Yokogawa CSU10), laser module with 491nm and 561nm excitation (Spectral Applied Research LMM2), and a C9100-13 EM-CCD camera (Hamamatsu). Image acquisition was carried out using the Volocity software (Perkin Elmer). A frame delay of 20s with 0.7μm Z-intervals was used to image mitotic spindles and centrosomes, while images were acquired every 30 s with 1μm Z-intervals for automatic tracking.

#### Analysis of the Orientation of Cell Division and of the Principal Axis of Cell Shapes in Live Embryos

To analyse the relationship between cell shape and cell division orientations, we imaged *ubi-DE-Cadherin-GFP/En>Venus; Jupiter::Cherry/+* embryos to label the cell membranes, the PSBs and the mitotic spindle, respectively. Live imaging on a spinning disk microscope was performed using a 100x objective to better visualise the mitotic spindle. 21 short movies (n=3 movies from stage 9 embryos, n=15 movies from stage 10 embryos, n=3 movies from stage 11 embryos) were analysed. The shape of dividing cells were analysed at t=-12 minutes from the end of cytokinesis, which corresponds roughly to the start of NEBD as described in the next paragraph. Cell shapes were traced manually in Fiji and their principal axis orientation as well as longest and shortest axes extracted using the best-fit ellipse tool. The angle of cell shape orientation was measured relative to the AP axis (α_Shape_), using PSBs as landmarks (see Figures S1A–S1B″). Cell elongation was measured by calculating the log_10_ of the ratio between the fitted ellipse’s longest and shortest axis, shortened as log_10_(long axis/short axis) on graphs. Cell division orientation relative to AP (α_Division_) at anaphase was obtained by measuring the angle between the mitotic spindle (for cells expressing Jupiter::mCherry, Figure 1F) or the two centrosomes (for cells expressing Asl-GFP, Figure 5) and the local PSB orientation (see above and Figures S1A–S1B″). The angular difference between the cell division orientation and the principal axis of shape is measured as |α_Shape_-α_Division_|. The rotation of the mitotic spindle was measured relative to AP, by measuring the angle between the spindle length and the local PSB orientation every 20 s from NEBD to cytokinesis (Figure 5C). NEBD was identified by inspecting the Jupiter::Cherry signal, since before NEBD, Jupiter is excluded from the nucleus.

#### Automated Tracking and Cell Shape versus Tricellular Vertex Analysis

In addition to the above manual analysis, cell shapes and tricellular vertex distributions were analysed using automated tracking of dividing cells (Figure 4). Embryos from the *sqh*^*AX3*^; *shotgun::GFP*, *sqh::mCherry* genotype (to label E-Cadherin and MRLC, respectively) were imaged from late stage 8 to stage 10 for 2 to 3 hours using a 40x/1.3 NA Nikon Plan Fluor objective. Cells were segmented and tracked in five movies with manually curated automated methods as previously described (Blanchard et al., 2009, Butler et al., 2009). Briefly, the movies’ *Z*-stacks were transformed into stacks of two-dimensional representations of curved surfaces, at successive depths from the surface of the embryo. The depth from the surface of the embryo which gave the clearest view of cell membranes was selected for tracking. Using an adaptive watershedding algorithm, the tracking programme identifies cells and links them iteratively. The software stores the coordinates of cell centroids and the pixelated shapes describing each cell, together with information concerning cell lineages.

The timepoint of the first cell division in the mesectoderm (at late stage 8) was used to synchronise movies. Only ventral neurectoderm cell divisions, which start at stage 9, were analysed. PSBs were identified by their enrichment of the MRLC-mCherry signal. Mother cells were automatically identified at the frame before abscission, when a new junction could be detected separating the mother into two daughter cells (*n*=359). Potential cell division events were further rejected if they were not also associated with the following behaviours: an approximate halving of mother cell area into daughters; an increase in cell area over the preceding 10 minutes; an increase in cell elongation and perimeter to area ratio in the preceding 3 minutes, due to anaphase cell elongation; the appearance of a Myosin-II cytokinesis ring and constriction to form a ‘dumbbell’ shape in the previous 1 minute, due to cytokinesis. A handful of remaining false positives (< 10) were manually excluded leaving *n*=359 cell division events for orientation analysis.

The orientation of cell division was measured as the orientation between daughter centroids at abscission(*θ*_division_). This and subsequent orientations were recorded as an angle relative to the AP axis orientation. For each mother cell, the principal axis of cell shape and the vertex distribution was analysed 12 minutes previously, corresponding to approximately to the start of NEBD. At this timepoint, the orientation (*θ*_shape_) and magnitude (*η*_shape_, between 0, if perfectly circular and 1, if infinitely stretched) of cell elongation and the orientation (*θ*_Vtx_) and magnitude (*η*_Vtx_, between 0, if uniformly distributed, and 1, if split into two diametrically opposed groups) of vertex clustering were calculated according to published methods (Bosveld et al., 2016). Briefly, *θ*_shape_ and *η*_shape_ were taken as the principal eigenvector and one minus the ratio of eigenvalues of a cell shape inertia matrix, respectively. The inertia matrix was calculated from the angular variation in length of all vectors from the cell centroid to perimeter pixels. *θ*_Vtx_ and *η*_Vtx_ were similarly taken from an inertia matrix, this time describing the orientation and strength of polarity of the distribution of tricellular vertices. This matrix was calculated from the angular variation in length of all vectors from the cell centroid to tricellular vertices. We then calculated the absolute difference in degrees between the orientation of cell division (*θ*_division_) and the orientations of both cell elongation (*θ*_shape_) and vertex clustering (*θ*_Vtx_) and compared these distributions.

#### Y-27632 Rho Kinase Inhibitor Injections

Stage 8 *sqh*^*AX3*^*/+; Asl-GFP/sqhGFP42; AslB46/GAP43-mCherry* embryos were mounted with their ventral side facing a glass coverslip with heptane glue, covered with halocarbon oil and injected through the posterior into the yolk at room temperature with 1 mM Y27632 (TOCRIS) in dH_2_O, or dH_2_O in control experiments (Monier et al., 2010). This low concentration of Y-27632 disrupts actomyosin contractility at the PSBs but does not affect cell division (Monier et al., 2010, Urbano et al., 2018). Note that (Chanet et al., 2017) employ a much higher concentration, 50mM, to disrupt cell division in *Drosophila* embryos. Embryos were allowed to recover for 30 minutes at 18°C, then imaged for ∼2 hours at 21°C.

#### Laser Ablation

Laser ablation experiments were performed using a TriM Scope II Upright 2-photon Scanning Fluorescence Microscope controlled by Inspector Pro software (LaVision Biotec) equipped with a tuneable near-infrared (NIR) laser source delivering 120 femtosecond pulses with a repetition rate of 80 MHz (Insight DeepSee, Spectra-Physics). The laser was set to 927nm, with power ranging between 1.40-1.70 W. The maximum laser power reaching the sample was set to 220 mW and an Electro-Optical Modulator (EOM) was used to allow microsecond switching between imaging and treatment laser powers. Laser light was focused by a 25x, 1.05 Numerical Aperture (NA) water immersion objective lens with a 2mm working distance (XLPLN25XWMP2, Olympus). Ablations were carried out during image acquisition (with a dwell time of 9.27 μsec per pixel), with the laser power switching between treatment and imaging powers as the laser scanned across the sample. Targeted line ablations of ∼2 μm length were performed at the centre of junctions at the PSBs or non-boundary dorsoventral (DV)-oriented junctions as control, using a treatment power of 220 mW. Images were acquired with a frame delay of 731 ms, more than 45 ablations per condition were carried out, 2-4 ablations per embryo. For consecutive ablations, line ablations of ∼2 μm length were performed at the centre of junctions at PSBs, and after 20 s a second ablation was carried out on the same PSB two vertices away from the first cut (see Figure 3A), as previously described (Rudolf et al., 2015). Images were acquired with a frame delay of 1s, more than 45 ablations per condition were carried out, 2-4 ablations per embryo. For loss of tension experiments, line ablations of ∼2 μm length were performed at the centre of junctions on a PSB next to a dividing cell in metaphase. Ablations were repeated every 25 seconds to prevent tissue healing, and after imaging kymographs were inspected to verify loss of recoil upon consecutive ablations (arrows, Figure S3J). Control ablations were carried out by setting the EOM unit treatment power at 25% of 220 mW (the same intensity used to image the sample) instead of 100%. As for treatment ablations, control ablations were also repeated. Images were acquired with a frame delay of 1s.

#### Laser Ablation Analysis

To analyse recoil velocities, images were background subtracted and denoised using Fiji. A kymograph spanning the ablated region was generated using the dynamic reslice function in Fiji, and the distance between the two ends of the cut was measured up to 20 seconds after ablation using a custom-made Matlab script (Curran et al., 2017). Linear regression was performed on the first 5 timepoints after ablation and the slope of the regressed line was used as a measure of the cut ends recoil velocity (Tetley et al., 2016). Junction length for PSB and non-PSB interfaces was measured using Fiji, and Myosin intensity was quantified in Fiji by drawing a 3 pixel-wide line selection on the junction of interest at t=0 and normalising it by dividing it by the signal of Myosin in the cytoplasm of the same cell at the same timepoint.

To measure Myosin signal intensity over time (Figures S2A, S3E, and 6B), a 3 pixel-wide line selection on the junction of interest or a ∼5μm diameter circle on the wounded/control area was drawn in Fiji and its intensity was measured and normalised by subtracting the mean grey value of the whole imaged area for each timepoint to correct for sample bleaching.

#### Laser Wounding

Circular ablations of ∼5μm diameter were performed twice with a 1 second interval on non boundary junctions next to a metaphase non-boundary cell, using an EOM treatment power of 80% of 220 mW. To measure tension upon wound healing, a line ablations of ∼2 μm length were performed at the centre the wounded area 90 seconds after the circular ablation, which corresponds to the peak of Myosin intensity (see Figure 6B) and the recoil speed was measured as described. Images were acquired with a frame delay of 2s.

#### Quantifications from Immunostainings

Quantifications were carried out on maximum intensity projections, which were derived from the minimum number of z-slices needed to contain all the signal. To quantify whether Pins or Mud were enriched at the PSB, the position of the PSB was identified by counterstaining with anti-En or anti-Wg antibodies. PSB or non-PSB interfaces were traced as 3-pixel wide lines and the fluorescence intensity of the selection was normalized to the modal grey value of the embryo (Urbano et al., 2018). To quantify the extent of Pins knockdown, control RNAi and Pins RNAi images were acquired in the same session using the same confocal settings. Maximum intensity projections were generated for each channel, the outline of the embryo was drawn and the absolute fluorescence intensity of the control channel (P-Tyrosine) or the experiment channel (Pins) was measured and plotted (Urbano et al., 2018).

#### Centrosome Tracking

Movies from H_2_O or Y27632-injected *sqh*^*AX3*^*/+; Asl-GFP/SqhGFP42; AslB46/GAP43-mCherry* embryos were analysed using the Imaris software (Bitplane). First, movies were corrected for rotational and translational xyz drift. Boundary cells were identified by inspection of the MRLC-GFP (*sqhGFP42*) signal and individual centrosomes were manually tracked along the 3 xyz dimensions from NEBD to cytokinesis. NEBD was identified by inspecting the MRLC-GFP signal, since before NEBD, Myosin is partially excluded from the nucleus(Barros et al., 2003). Measurements such as centrosome average speed, total distance travelled, total displacement and persistence (displacement divided by total distance travelled) were calculated in Imaris, exported and analysed using Excel (Microsoft) or GraphPad Prism.

### Quantification and Statistical Analysis

Statistical analysis was performed using GraphPad Prism. Angular histograms were plotted using a custom-made R script. Data from quantifications are reported as mean±SD, mean±SEM, median±25^th^/75^th^ percentiles or histograms according to whether they follow a normal distribution or not. On normally distributed data, two-tailed Student’s t-tests (two experimental groups) or One-way Anova followed by Dunnett’s multiple comparisons test (multiple experimental groups) were performed, while for non-normally distributed datasets Mann-Whitney, Kolmogorov-Smirnov (two experimental groups) or Kruskal-Wallis multiple comparisons tests (multiple experimental groups) were performed as described in the figure legends. Statistical analysis of cell division orientation histograms was carried out using two-tailed Mann-Whitney non-parametric tests (Luxenburg et al., 2011, Williams et al., 2011). For all tests, a confidence level of 0.05 was considered statistically significant.

### Data and Software Availability

Raw dataset spreadsheets are available on Mendeley Data: https://doi.org/10.17632/r4tdprzd8v.1.

## Supplemental Information

Document S1. Figures S1–S6

Document S2. Article plus Supplemental Information
