# Supplementary material for: Actomyosin-Driven Tension at Compartmental Boundaries Orients Cell Division Independently of Cell Geometry In Vivo
Source: Dev Cell. 2018 Dec 17;47(6):727–740.e6. doi: 10.1016/j.devcel.2018.10.029 (PMC6302072; doi:10.1016/j.devcel.2018.10.029)
Supplement: Document S1. Figures S1–S6 [file mmc1.pdf]

Developmental Cell, Volume *47*

Supplemental Information

Actomyosin-Driven Tension
at Compartmental Boundaries Orients Cell Division
Independently of Cell Geometry *In Vivo*

Elena Scarpa, Cédric Finet, Guy B. Blanchard, and Bénédicte Sanson

Figure S1

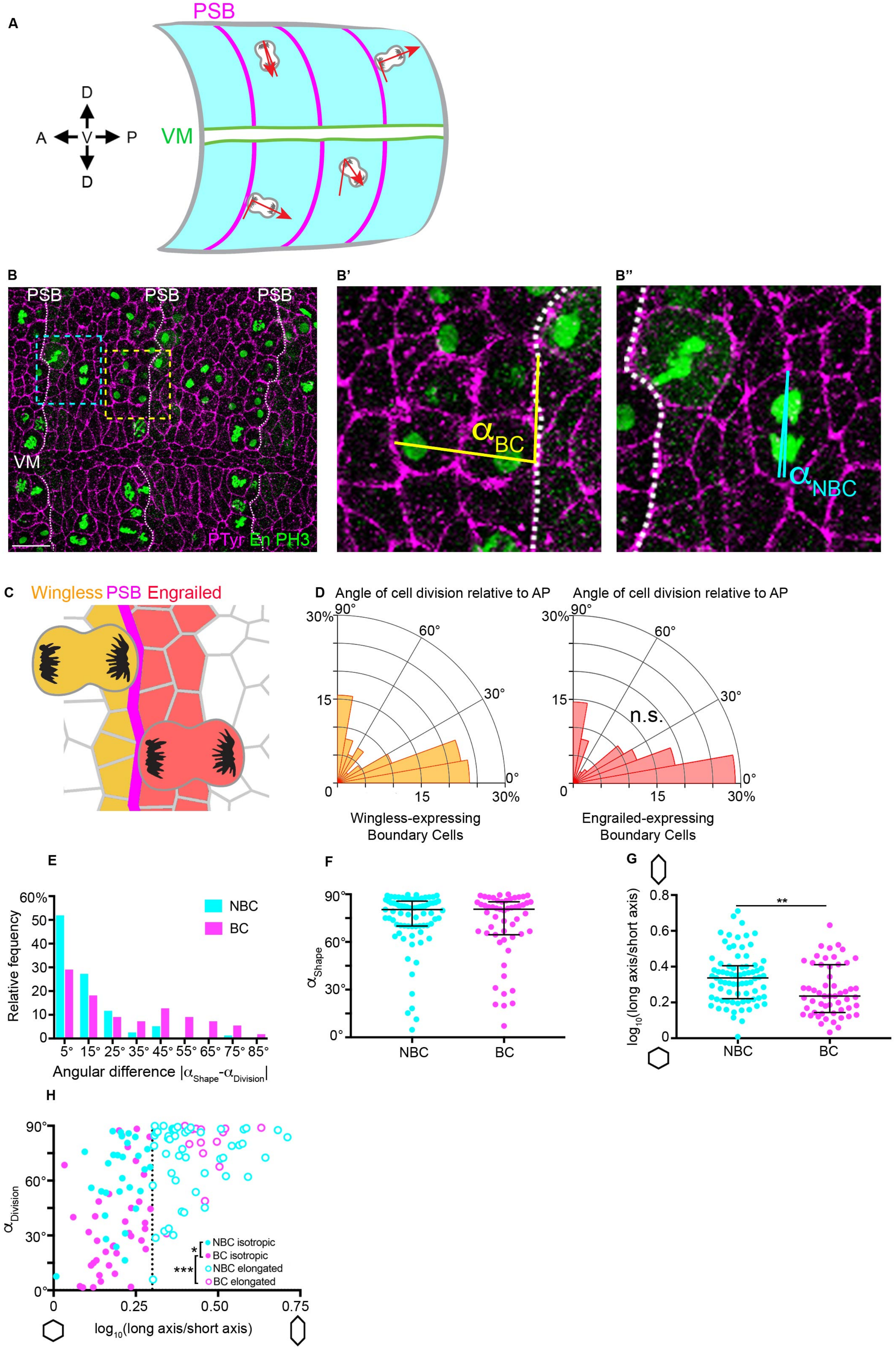
A
PSB
D
A
V
P
D
VM
B
PSB
PSB
PSB
VM
PTyr En PH3
B’
αBC
B”
αNBC
C
Wingless PSB Engrailed
D
Angle of cell division relative to AP
Angle of cell division relative to AP
n.s.
Wingless-expressing Boundary Cells
Engrailed-expressing Boundary Cells
E
NBC
BC
Relative fequency
Angular difference |αShape-αDivision|
F
αShape
NBC
BC
G
log10(long axis/short axis)
**
NBC
BC
H
αDivision
NBC isotropic
BC isotropic
NBC elongated
BC elongated
log10(long axis/short axis)

Figure S1, related to Figure 1

(A) Diagram illustrating how the orientation of cell division is measured in fixed embryos. The position of the parasegmental boundaries (PSBs) is determined by staining embryos with either Wingless or Engrailed (see diagram in e). Throughout the manuscript, the division angles are given relative to the orientation of the embryo's antero-posterior (AP) axis. Although the ventral midline (VM) is a good read-out of the AP axis orientation in the embryo, the division angles were instead measured systematically relative to the curvature of the nearest PSBs, for both BC and NBC, as this allows to correct for tissue deformation due to embryo mounting. These angles are then converted by 90 degrees to obtain the orientation of cell division with respect to the AP axis. (B) Example of an immunostaining used for angle measurements. A maximum projection of a confocal stack of a stage 9 embryo immunostained for phospho-Histone H3 (to highlight the chromosomes in dividing cells), Engrailed (to find the PSBs) and phospho-Tyrosine (to label the cell shapes) is shown. Note that the angle of cell division is measured in anaphase or telophase cells because by then the spindles have finished rotating (see Fig. 5). PSBs are highlighted by a dashed line. VM, ventral midline. Scale bar 20 μm. (B') Close up of a BC (at telophase), with the division angle measured relative to the orientation of the PSB. (B'') Close-up of a NBC (at anaphase), with the division angle measured relative to the nearby PSB. (C,D) The orientation of dividing boundary cells was analysed according to their position on either side of the PSB, either anteriorly or posteriorly, in Wingless (Wg) or Engrailed (En)-expressing boundary cells, respectively. No significant difference was found between the two populations (Wg-positive, n=165; En-positive, n=124; Mann-Whitney test, U=9569, P=0.347). (E) Histogram of the angular differences between the orientation of cell division and the orientation of interphase cell shape for NBC (blue) and BC (pink) (see corresponding cumulative histogram in Fig. 1i). This angular difference is small for NBCs and large for BCs. (F) In average, the principal axis of interphase cell shape is oriented perpendicular to the AP axis of the embryo for both NBC and BC populations (NBC, n=77; BC, n=55; Mann-Whitney test, U=2007, P=0.613; Median ± interquartile range shown). (G) BC have slightly less elongated shapes than NBC (NBC, n=77; BC, n=55; Mann-Whitney test, U=1487, **P=0.003;. Median ± interquartile range shown). (H) Cell division orientation relative to AP as a function of $\log_{10}$(long axis/short axis). For elongated cells (above 0.3, long axis/short axis ratio of 2), both NBC and BC behave similarly, dividing perpendicular to AP (NBC, n=48; BC, n=16; Kruskal-Wallis test, H=45.65, P=0.6393). However, for isotropic or moderately elongated cells (below 0.3), NBC and BC behaviours are significantly different (NBC, n=29; BC, n=39; Kruskal-Wallis test, H=45.65, *P=0.0194). For BC, the angle of cell division between elongated and isotropic cells was significantly different (Elongated BC, n=16; Isotropic BC, n=39; Kruskal-Wallis test, H=45.65, ***P<0.0001).

Figure S2

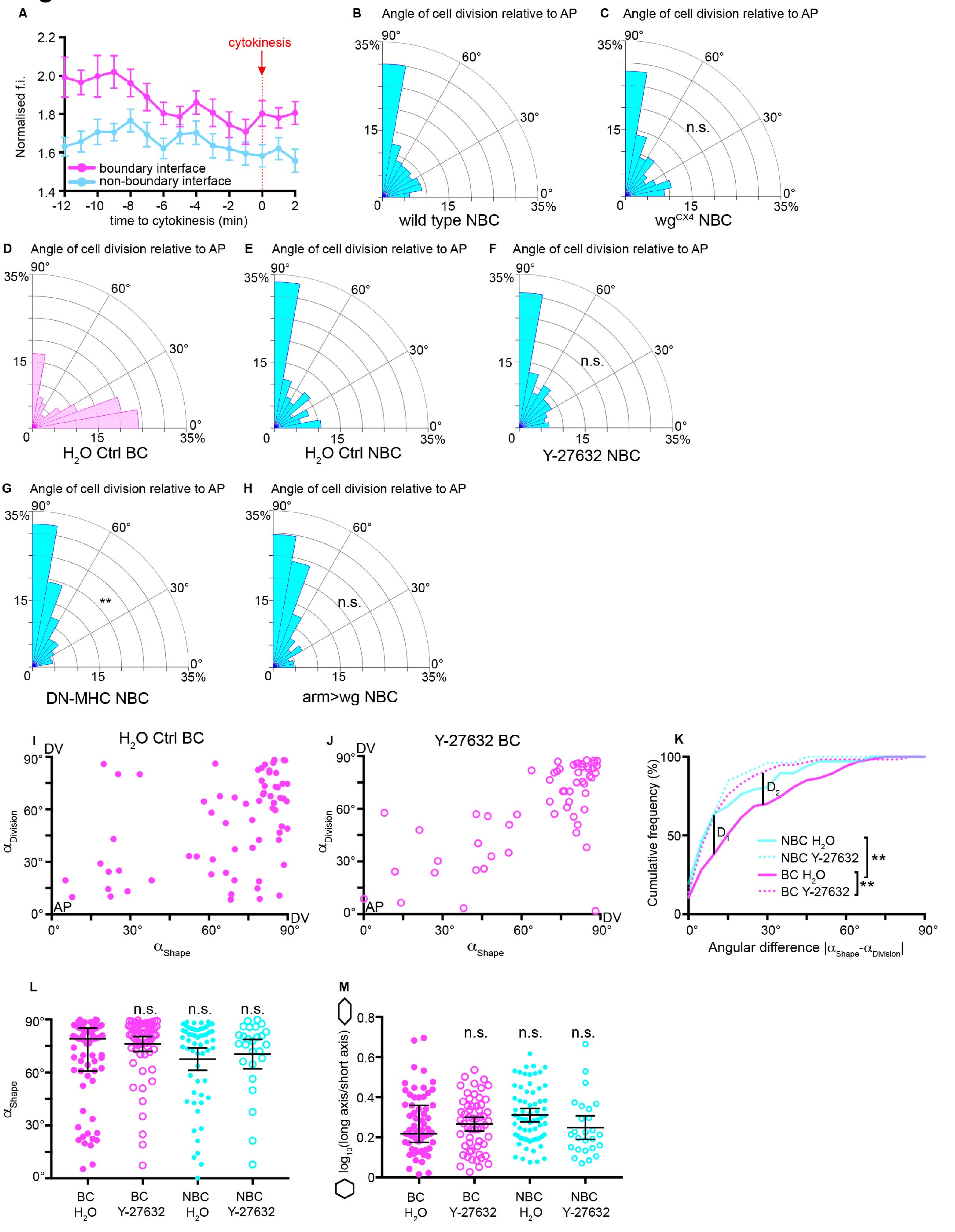

A
cytokinesis
Normalised f.i.
boundary interface
non-boundary interface
time to cytokinesis (min)
B Angle of cell division relative to AP
wild type NBC
C Angle of cell division relative to AP
n.s.
wg^{CX4} NBC
D Angle of cell division relative to AP
H_2O Ctrl BC
E Angle of cell division relative to AP
H_2O Ctrl NBC
F Angle of cell division relative to AP
n.s.
Y-27632 NBC
G Angle of cell division relative to AP
**
DN-MHC NBC
H Angle of cell division relative to AP
n.s.
arm>wg NBC
I H_2O Ctrl BC
DV
AP
$\alpha_{Division}$
α_{Shape}
J Y-27632 BC
DV
AP
$\alpha_{Division}$
α_{Shape}
K
Cumulative frequency (%)
D_1
D_2
NBC H_2O
NBC Y-27632
BC H_2O
BC Y-27632
**
**
Angular difference $|\alpha_{Shape}-\alpha_{Division}|$
L
α_{Shape}
n.s.
n.s.
n.s.
BC H_2O
BC Y-27632
NBC H_2O
NBC Y-27632
M
$\log_{10}$(long axis/short axis)
n.s.
n.s.
n.s.
BC H_2O
BC Y-27632
NBC H_2O
NBC Y-27632

Figure S2, related to Figure 2

(A) MRLC normalised fluorescence intensity for boundary and non-boundary interfaces of dividing BCs throughout mitosis (*n*=24 cells). (B-C) Cell division angles relative to AP for NBC in: (B) wild type (*n*=589) and (C), wg^{CX4} (*n*=618) for stages 9 to 11 embryos (Mann-Whitney test, *U*=128400, P=0.272). (D-F) Cell division angles relative to AP in embryos injected with H_2O, BC (c, *n*=83) and NBC (d, *n*=132), and in embryos injected with the ROK inhibitor Y-27632, NBC (e, *n*=117) (Kruskal-Wallis tests, H_2O BC vs H_2O NBC, *H*=24.28, P<0.0001; H_2O NBC vs Y-27632 NBC, *H*=24.28, P=1.0). (G) Cell division angles relative to AP for NBC in embryos expressing DN-MHC (*n*=454; wild type *n*=391 (from Fig. 1d); Mann-Whitney test, *U*=78689, **P=0.0044). (H) Cell division angles relative to AP for NBC in embryos overexpressing Wingless (*arm>wg)* (*n*=212; *n*=391, wild type (from Fig. 1); Kruskal-Wallis test, *H*=211.2, P=0.091). (I,J) Correlation between angles of cell division and cell shape: BC from embryos injected with Y-27632 follow the long axis rule better than BC from H_2O injected embryos (*n*=58; Spearman's rho test, *r*=0.62, P<0.001 and *n*=67; Spearman's rho test, *r*=0.42, P<0.001, respectively). (K) Cumulative histogram of the angular difference between cell division and interphase cell shape for BC H_2O (*n*=67), BC Y27632 (*n*=58), NBC H_2O (*n*=67), NBC Y-27632 (*n*=26). H_2O BC are significantly different from H_2O NBC (Kolmogorov-Smirnov test, D_1=0.29, **P=0.0051). H_2O BC significantly different from Y-27632 BC, with a higher proportion of cells dividing according to their interphase shape (Kolmogorov-Smirnov test, D_2=0.32, **P=0.0031). All other comparisons are not significantly different. (L) Cell shape principal axis orientation for H_2O or Y-27632 treated BC and NBC (BC H_2O, *n*=67; BC Y27632, *n*=58; NBC H_2O, *n*=67; NBC Y27632, *n*=26). Kruskal-Wallis tests, all comparisons are not significantly different. Median ± interquartile range shown. (M) Cell elongation, measured as $\log_{10}$(long axis/short axis) for H_2O or Y-27632 treated BC and NBC (BC H_2O, *n*=67; BC Y27632, *n*=58; NBC H_2O, *n*=67; NBC Y27632, *n*=26). Kruskal-Wallis tests, all comparisons are not significantly different. Median ± interquartile range shown.

Figure S3

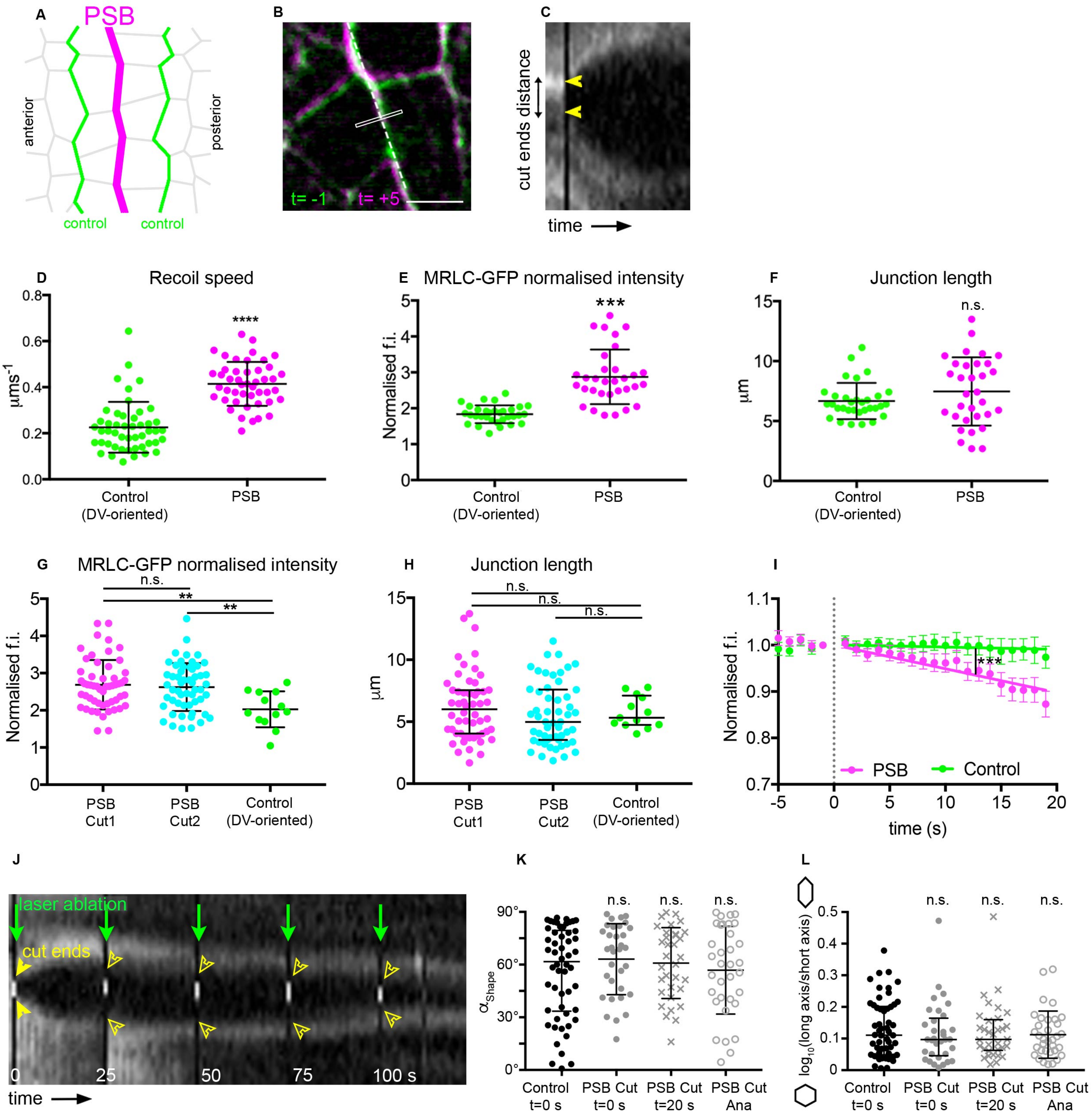
A
PSB
anterior
posterior
control
control
B
t= -1
t= +5
C
cut ends distance
time
D
Recoil speed
μms-1
****
Control
(DV-oriented)
PSB
E
MRLC-GFP normalised intensity
Normalised f.i.
***
Control
(DV-oriented)
PSB
F
Junction length
μm
n.s.
Control
(DV-oriented)
PSB
G
MRLC-GFP normalised intensity
Normalised f.i.
n.s.
**
**
PSB
Cut1
PSB
Cut2
Control
(DV-oriented)
H
Junction length
μm
n.s.
n.s.
n.s.
PSB
Cut1
PSB
Cut2
Control
(DV-oriented)
I
Normalised f.i.
***
PSB
Control
time (s)
J
laser ablation
cut ends
0
25
50
75
100 s
time
K
αShape
n.s.
n.s.
n.s.
Control
t=0 s
PSB Cut
t=0 s
PSB Cut
t=20 s
PSB Cut
Ana
L
log10(long axis/short axis)
n.s.
n.s.
n.s.
Control
t=0 s
PSB Cut
t=0 s
PSB Cut
t=20 s
PSB Cut
Ana

Figure S3, related to Figure 3.

(A-D) Tension is higher at PSB compared to non-boundary interfaces of boundary cells. (A) Tension at the PSB (magenta) is compared to that of adjacent control non-boundary interfaces (green) in MRLC-GFP expressing embryos. (B) Overlay of a PSB before (-1 second, green) and after (+5 seconds, magenta) laser ablation (white rectangle, cut site). Scale bar 5 µm. (C) Kymograph spanning the dashed line in (B) used to measure the distance between cut ends over time (arrowheads). (D) Speed of recoil upon ablation (PSB, *n*=48; Control, n=46; Mann-Whitney test, *U*=209.5, ****P<0.0001). Mean ± SD shown. (E) Normalised Myosin II fluorescence intensity for ablated control and PSB interfaces (PSB, *n*=48; Control, *n*=46; Student's t-test, *t*=7.35, ***p<0.0001). Mean ± SD shown. (F)Junction length of ablated control and PSB interfaces (PSB, *n*=48; Control, *n*=46; Mann-Whitney test, *U*=442, P=0.351). Mean ± SD shown. (G) Normalised Myosin II fluorescence intensity at t=0, for ablated control (non-PSB DV-oriented junctions) and PSB interfaces for each ablation site (PSB Cut1, *n*=52; PSB Cut2, *n*=52; Control, *n*=29; One-way Anova, *F*=5.743, P=0.0042; Sidak's multiple comparison test for PSB Cut1 vs Control, **P=0.0033; for PSB Cut2 vs Control, **P=0.0092; for PSB Cut1 vs PSB Cut2 P=0.9396) Means ± SDs shown. (H) Junction length of ablated control and PSB interfaces for consecutive PSB cuts (PSB Cut1, *n*=52; PSB Cut2, *n*=52; Control, n=29; Kruskal-Wallis tests; PSB Cut1 vs Control, *H*=1.68, P>0.9999; PSB Cut1 vs PSB Cut2, P=0.6418). Median ± interquartile range shown. (I) Myosin intensity is decreased at the actomyosin cable after PSB ablation, compared to control DV-oriented interfaces anterior or posterior to the ablated region. Measurements are normalised to the initial fluorescence intensity before ablation. Means ± SEM shown. Slopes are significantly different (ANCOVA on linear regression, *F*=130, ***P<0.0001). (J) A representative kymograph for a PSB-cut experiment. Laser ablation was repeated every 25 s to prevent wound healing (green arrows). Lack of recoil upon repeated cuts was used to estimate successful loss of tension at the PSB (compare curve highlighted by the full yellow arrowhead, which shows recoil of cut ends, with the hollow yellow arrowheads, which show lack of recoil). (K) Orientation of the cell shape principal axis for control and PSB-cut treated dividing cells at t=0s, and after ablation, at t=20s (metaphase) or at anaphase onset (Control, *n*=54; PSB-cuts, *n*=33; Kruskal-Wallis tests, *H*=1.332, all pair-wise comparisons P>0.99). Medians ± interquartile ranges shown. (L) Cell shape $\log_{10}$-ratios for control and PSB-cut treated cells at t=0s, and after ablation, at t=20s (metaphase) or at anaphase onset (Control, *n*=54; PSB-cuts, *n*=33; Kruskal-Wallis test, *H*=0.941, all pair-wise comparisons P>0.99). Medians ± interquartile ranges shown.

Figure S4

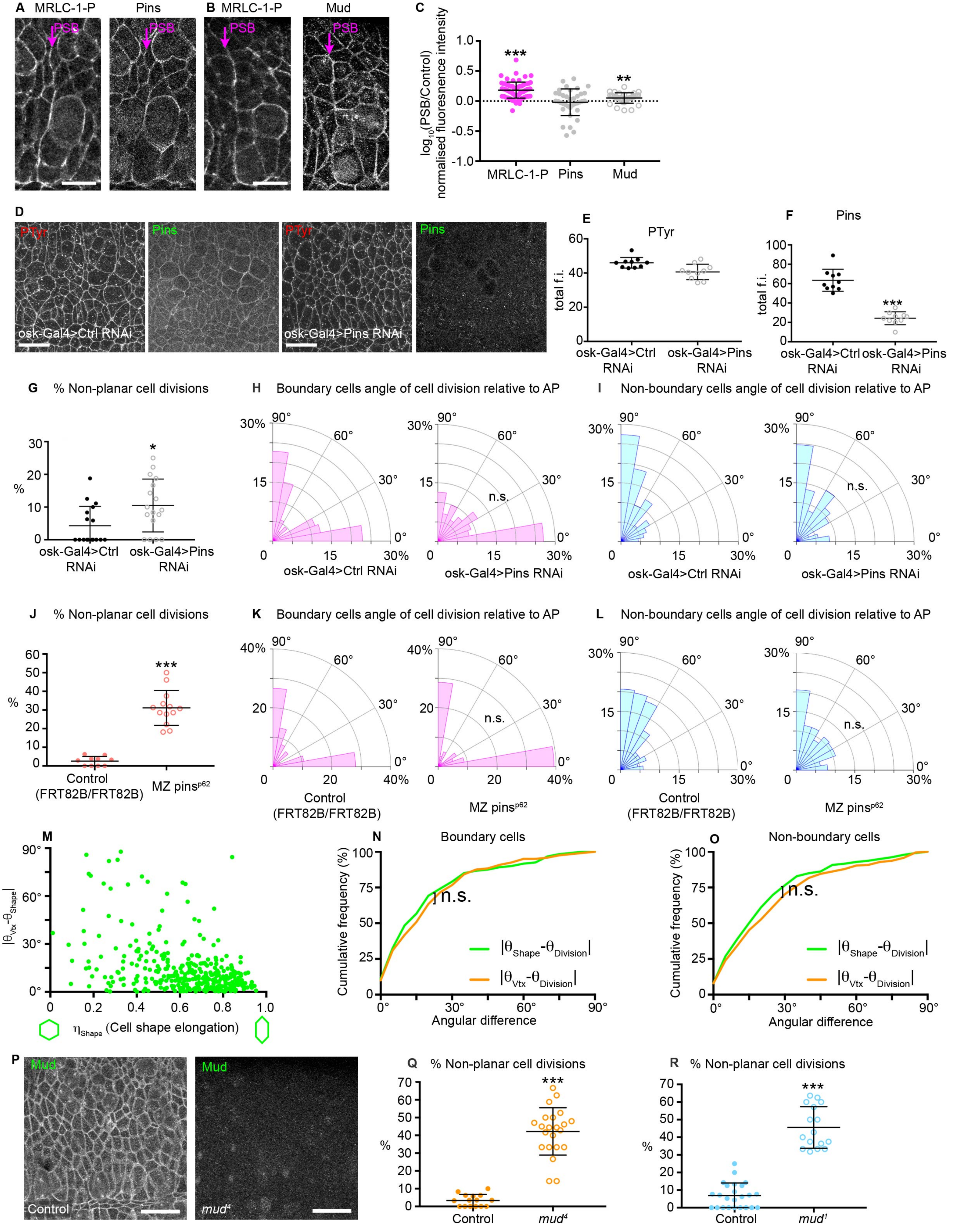

A MRLC-1-P
Pins
B MRLC-1-P
Mud
PSB
C
log10(PSB/Control) normalised fluoresnence intensity
***
**
MRLC-1-P
Pins
Mud
D
PTyr
Pins
osk-Gal4>Ctrl RNAi
osk-Gal4>Pins RNAi
E
PTyr
total f.i.
F
Pins
***
G
% Non-planar cell divisions
*
H
Boundary cells angle of cell division relative to AP
n.s.
I
Non-boundary cells angle of cell division relative to AP
J
% Non-planar cell divisions
***
Control (FRT82B/FRT82B)
MZ pins^p62
K
Boundary cells angle of cell division relative to AP
L
Non-boundary cells angle of cell division relative to AP
M
|θVtx-θShape|
ηShape (Cell shape elongation)
N
Boundary cells
Cumulative frequency (%)
Angular difference
|θShape-θDivision|
|θVtx-θDivision|
O
Non-boundary cells
P
Mud
Control
mud4
Q
% Non-planar cell divisions
Control
mud4
R
% Non-planar cell divisions
Control
mud1

Figure S4, related to Figure 4

(A-C) Endogenous MRLC-1P and Pins localization in wild type embryos (A). Endogenous MRLC-1P and Mud localization in wild type embryos (B). (C) Quantitation of Myosin-1-P, Mud and Pins fluorescence intensity enrichment at boundary interfaces compared to non-boundary interfaces (*n*=71, Myo-1-P interfaces; *n*=37, Pins; *n*=34, Mud; Wilcoxon signed-rank tests; MRLC-1P, ***P<0.0001; Pins, P=0.988; Mud, **P=0.0019). Means ± SDs shown. (D-F) Endogenous Pins and PTyr immunostainings for Control RNAi and Pins RNAi embryos. Scale bar 20 μm (D). Quantitation of PTyr (E) and Pins (F) total fluorescence intensity (*n*=10 embryos, Ctrl RNAi; *n*=10 embryos, Pins RNAi; unpaired Student's *t*-tests; PTyr, *t*=3.09, P=0.0064; Pins, *t*=9.40, ***P<0.0001). Means ± SDs shown. (G) Percentage of cells dividing out of the epithelial plane for control (9/201 cell divisions) or Pins (21/177 cell divisions) RNAi (*n*=17 embryos per genotype, Mann-Whitney test, *U*=80.5, *P=0.0216). Means ± SDs shown. (H) Histogram of cell division orientation for boundary cells (BC) in Pins RNAi (*n*=111), or control RNAi (*n*=105; Mann-Whitney test, *U*=5323, P=0.272) embryos. (I) Histogram of cell division orientation for non-boundary cells in Pins RNAi (*n*=178), or control RNAi (*n*=201; Mann-Whitney test, *U*=16482, P=0.186) embryos. (J) Percentage of cells dividing out of the epithelial plane for FRT82B/FRT82B control (7/239 cell divisions) or *MZ* $Pins^{p62}$ (60/199 cell divisions) embryos (*n*=13 embryos, *MZ* $Pins^{p62}$; *n*=24 embryos, FRT82B/FRT82B control; Student's *t*-test, *t*=9.349, ***P<0.0001). Means ± SDs shown. (K) Histogram of cell division orientation for boundary cells (BC) in *MZ* $Pins^{p62}$ mutant embryos (*n*=56 cells), or FRT82B/FRT82B control embryos (*n*=75 cells; Mann-Whitney test, *U*=1882, P=0.312) embryos. (L) Histogram of cell division orientation for non-boundary cells (NBC) in *MZ* $Pins^{p62}$ mutant embryos (*n*=112 cells), or FRT82B/FRT82B control embryos (*n*=155 cells; Mann-Whitney test, *U*=7489, P=0.056) embryos. (M) Scatterplot of the absolute difference between cell shape principal axis orientation and vertex cluster orientation, $|\theta_{Vtx}-\theta_{Shape}|$, against cell elongation (η_{Shape}). Note that less elongated cells have a higher angular difference between shape and vertex orientation (*n*=335 from 5 embryos). (N-O) Cumulative histograms of the Shape deviation angle or Vertex deviation angle for BC (a, *n*=120 from 5 embryos; Kolmogorov-Smirnov test, *D*=0.091, P=0.695) and NBC (b, *n*=239 from 5 embryos; Kolmogorov-Smirnov test, *D*=0.1046, P=0.1463). (P) Endogenous Mud immunostaining for control and mud^4 embryos. Scale bar 20 μm. (Q) Percentage of cells dividing out of the epithelial plane for control (12/277 cell divisions) or mud^4 (124/289 cell divisions) embryos (*n*=15 embryos, mud^4; *n*=22 embryos, control; Student's *t*-test, *t*=11.01, ***P<0.0001). Means ± SDs shown. (R) Percentage of cells dividing out of the epithelial plane for control (20/298 cell divisions) or mud^1 (81/181 cell divisions) embryos (*n*=16 embryos, mud^1; *n*=24 embryos, control; Student's *t*-test, *t*=12.94, ***P<0.0001). Means ± SDs shown.

Figure S5

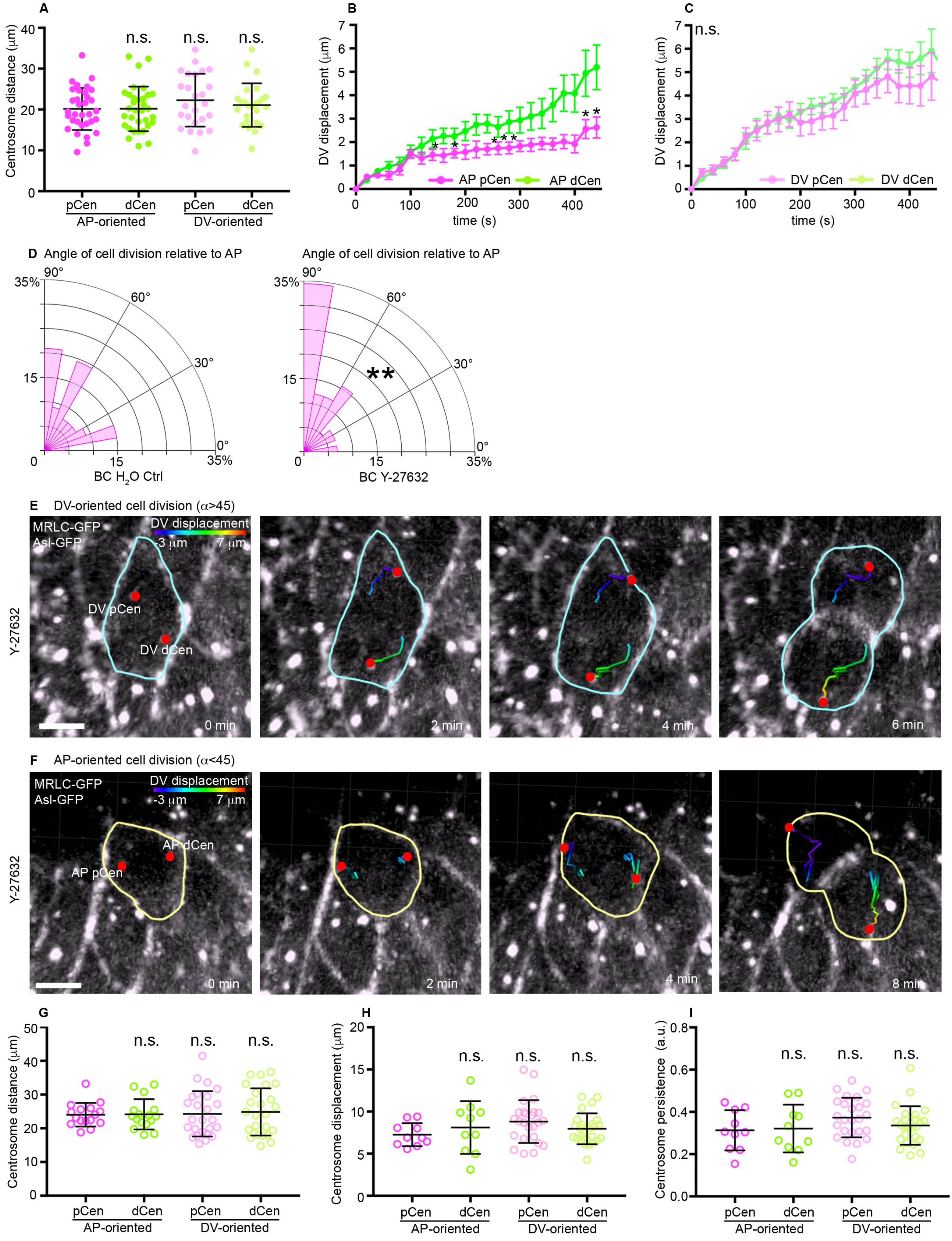

A
Centrosome distance (μm)
n.s.
n.s.
n.s.
pCen
dCen
pCen
dCen
AP-oriented
DV-oriented
B
DV displacement (μm)
time (s)
AP pCen
AP dCen
C
n.s.
DV displacement (μm)
time (s)
DV pCen
DV dCen
D Angle of cell division relative to AP
BC H2O Ctrl
Angle of cell division relative to AP
BC Y-27632
E DV-oriented cell division (α>45)
MRLC-GFP
Asl-GFP
DV displacement
-3 μm
7 μm
DV pCen
DV dCen
Y-27632
0 min
2 min
4 min
6 min
F AP-oriented cell division (α<45)
MRLC-GFP
Asl-GFP
DV displacement
-3 μm
7 μm
AP dCen
AP pCen
Y-27632
0 min
2 min
4 min
8 min
G
Centrosome distance (μm)
H
Centrosome displacement (μm)
I
Centrosome persistence (a.u.)
n.s.
pCen
dCen
pCen
dCen
AP-oriented
DV-oriented

Figure S5, related to Figure 5

(A) Total distance travelled by each centrosome from NEBD to cytokinesis (*n*=33, AP pCen; *n*=33, AP dCen; *n*=25, DV pCen; *n*=25, DV dCen; Kruskal Wallis tests, *H*=2.904, all pair-wise comparisons P>0.75). Means ± SDs shown. (B) Absolute displacement along the DV axis of the embryo over time (t=0 NEBD) for AP-Oriented BC divisions (*n*=16, AP pCen; *n*=16, AP dCen; paired Student's t-tests, P<0.05 shown by asterisk). Means ± SEM shown. (C) Absolute displacement along the DV axis of the embryo over time (t=0 NEBD) for DV-Oriented BC divisions (*n*=19, DV pCen; *n*=19, DV dCen; paired Student's t-tests, P>0.05). Means ± SEM shown. (D) Angles of division relative to AP for BC from embryos expressing MRLC-GFP and Asl-GFP injected with either H_2O or Y-27632 (*n*=67, BC H_2O; *n*=58, BC Y-27632; Mann-Whitney test, *U*=1525, **P=0.0384). (E,F) Representative DV-oriented (E) and AP-oriented (F) BC cell division from an Y-27632 injected embryo expressing MRLC-GFP and Asl-GFP. Centrosome tracks are highlighted and colour-coded for DV displacement. pCen and dCen, centrosomes proximal and distal from PSB, respectively. Scale bar 5 µm. (G) Total distance travelled by each centrosome from NEBD to cytokinesis for Y-27632 injected embryos (*n*=10, AP pCen; *n*=10, AP dCen; *n*=25, DV pCen; *n*=25, DV dCen; One-way Anova, F=0.0813, P=0.97, all pair-wise comparisons P>0.90). Means ± SDs shown. (H) Total displacement for each centrosome from NEBD to cytokinesis for Y-27632 injected embryos. (*n*=10, AP pCen; *n*=10, AP dCen; *n*=25, DV pCen; *n*=25, DV dCen; One-way Anova, *F*=1.278, P=0.28, all pair-wise comparisons P>0.15). Means ± SDs shown. (I) Persistence for each centrosome from NEBD to cytokinesis for Y-27632 injected embryos (*n*=10, AP pCen; *n*=10, AP dCen; *n*=25, DV pCen; *n*=25, DV dCen; Kruskal Wallis tests, all pair-wise comparisons P>0.30). Means ± SDs shown.

Figure S6

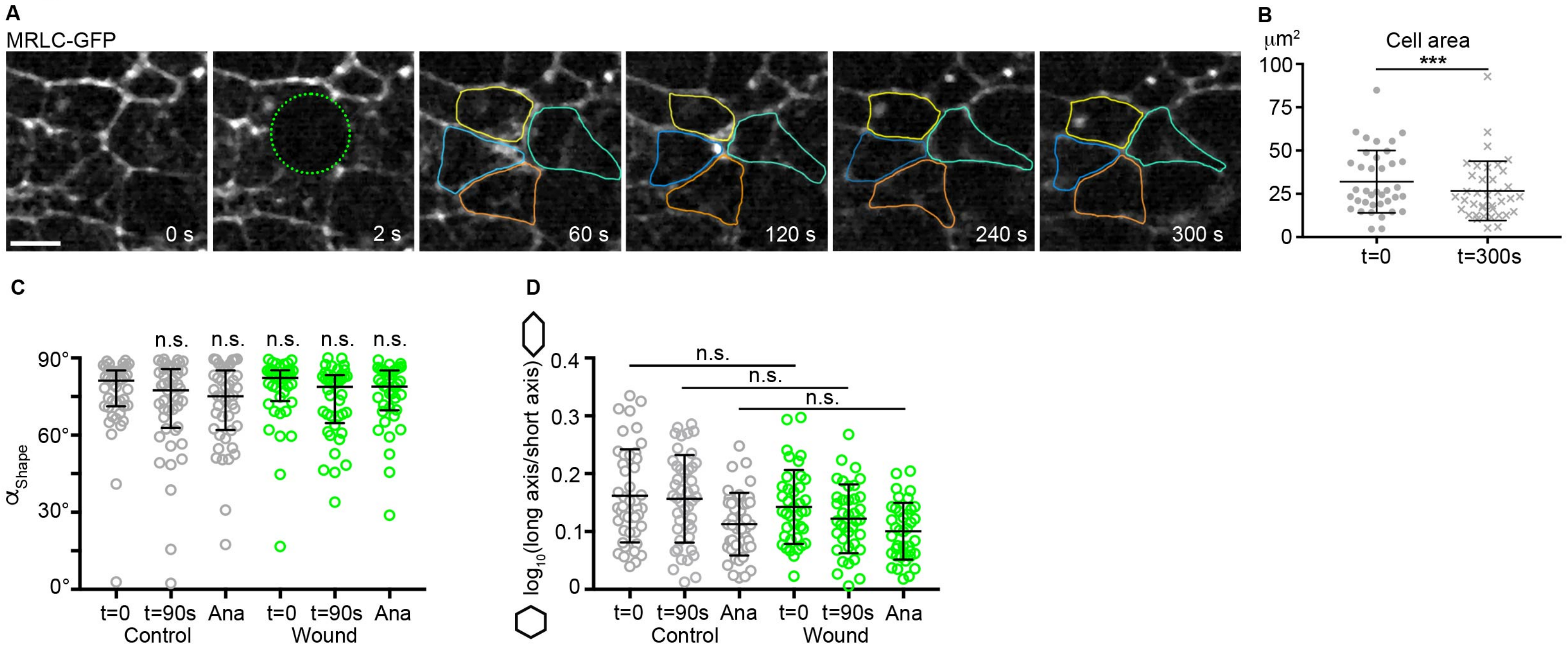
A
MRLC-GFP
0 s
2 s
60 s
120 s
240 s
300 s
B
μm2
Cell area
***
100
75
50
25
0
t=0
t=300s
C
n.s.
90°
60°
30°
0°
αShape
t=0
t=90s
Ana
Control
Wound
D
log10(long axis/short axis)
0.4
0.3
0.2
0.1
0

Figure S6, related to Figure 6

(A-B) Laser irradiation causes wound healing without triggering cell delamination. (A) Still images from a gain of tension experiment. Green circle: site of wounding. Cell outlines are highlighted, scale bar 5 µm. (B) Cell area at t=0 and at t=300s after wounding (*n*=38; Wilcoxon matched pairs signed-rank test, ***P=0.001). Means ± SDs shown. Although the area of the cells decreases a little after wounding, the cells do not delaminate. (C) Orientation of the cell shape principal axis for control and wound treated cells at t=0, t=90 seconds (metaphase) and anaphase onset after treatment (*n*=45 control, *n*=40 wound; Kruskal-wallis tests, all pair-wise comparisons P>0.5). Means ± interquartile ranges shown. (D) Cell elongation, measured as $\log_{10}$(long axis/short axis), for control and wound treated cells at t=0, at t=90s (metaphase) and anaphase onset after treatment (*n*=45, control; *n*=40, wound; One-way Anova, *F*=5.989, P<0.001; Sidak's multiple comparisons, all pair-wise comparisons P>0.05). Means ±SD shown. C and D shows that the geometry of the cells is not changed significantly by the wounding experiment.
